# Understanding the high electronic quantum similarity of a series of ligands used as inhibitors of the SARS-CoV-2 virus by molecular mechanics and density functional theory approaches

**DOI:** 10.12688/f1000research.127061.2

**Published:** 2024-02-06

**Authors:** Alejandro Morales-Bayuelo, Jesús Sánchez-Márquez

**Affiliations:** 1 Process Research Center of Tecnológico Comfenalco (CIPTEC), Industrial Engineering Program, Fundacion Universitaria Tecnologico Comfenalco, Cartagena, Colombia; 2 Department of Chemistry-Physics, Science Faculty, Río San Pedro University Campus, Cádiz University, Cádiz, Spain

**Keywords:** RNA dependent RNA polymerase SARS-CoV-2 virus, COVID-19 treatments, molecular docking, molecular quantum similarity, chemical reactivity descriptors, density functional theory.

## Abstract

**Background:**

A coronavirus identified in 2019, SARS-CoV-2, has caused a pandemic of respiratory illness, called COVID-19. Most people with COVID-19 experience mild to moderate symptoms and recover without the need for special treatments. The SARS‑CoV‑2 RNA‑dependent RNA polymerase (RdRp) plays a crucial role in the viral life cycle. The active site of the RdRp is a very accessible region, so targeting this region to study the inhibition of viral replication may be an effective therapeutic approach. For this reason, this study has selected and analysed a series of ligands used as SARS-CoV-2 virus inhibitors, namely: Darunavir (Daru), Dexamethasona (Dexame), Dolutegravir (Dolu), Fosamprenavir (Fosam), Ganciclovir (Gan), Insoine (Inso), Lopinavir (Lop), Ritonavir (Rito) and Tipranavir (Tipra).

**Methods:**

These ligands were analyzed using molecular docking, molecular quantum similarity using four similarity indices like overlap, Coulomb and their Euclidean distances. On the other hand, these outcomes were supported with chemical reactivity indices defined within a conceptual density functional theory framework.

**Results:**

The results show the conformations with the highest root-mean-square deviation (RMSD), have π-π stacking interaction with residue LYS621, ARG555 and ASP623, CYS622, ASP760, among others. In the molecular quantum similarity, the highest indices have been obtained in the electronic similarity in comparison with the structural similarity.

**Conclusions:**

These studies allow the identification of the main stabilizing interactions using the crystal structure of SARS‑CoV‑2 RNA‑dependent RNA polymerase. In this order of ideas, this study provides new insights into these ligands that can be used in the design of new COVID-19 treatments. The studies allowed us to find an explanation supported in the Density Functional Theory about the chemical reactivity and the stabilization in the active site of the ligands.

## Introduction

Coronaviruses are the family of viruses to which SARS-CoV-2 belongs, the virus causing the COVID-19 pandemic. They were discovered in the 1960s, but their origin is still unknown. ^1^ Their different types cause different illnesses, from a cold to severe respiratory illness (a severe form of pneumonia). Most coronaviruses are not dangerous and can be treated effectively. In fact, most people contract a coronavirus at some point in their lives, usually during their childhood. ^1^ Although they are more frequent in autumn or winter, they can be found at any time of the year. ^1^^–^^3^

The coronavirus owes its name to the appearance it presents, since it is very similar to a crown or a halo. It is a type of virus present mainly in animals, but also in humans. In recent years, ^4^ three major epidemic outbreaks caused by new coronaviruses have been described. COVID-19/SARS-CoV-2: at the end of December 2019, ^4^^,^^5^ the first cases of a new coronavirus were reported in the city of Wuhan (China). Since then, the increase in new infections with the SARS-CoV-2 virus (initially called 2019nCoV), which causes the disease called COVID-19, has been continuous and its transmission from person to person has accelerated. ^5^ Reported cases already far exceed those of the 2002-2003 SARS epidemic. ^4^^,^^5^ SARS’s fatality rate is lower than that of other coronaviruses, but many more deaths are occurring (there are already more than 5 million, because the infected people number in the hundreds of millions worldwide (almost 300 million in early January 2022). ^4^^–^^6^

There is no specific treatment for SARS-CoV-2, but multiple drugs, alone or in combination, are being investigated, as well as the use of plasma from patients who have recovered. ^6^^–^^8^ The usefulness of the other drugs, which are being administered to patients in clinical trials or for compassionate use, are being studied. For example, Remdesivir is an antiviral drug that was initially developed for the disease caused by the Ebola virus but has also shown in vitro activity against SARS-CoV-2. ^9^ However, the results of this treatment have not been as satisfactory as expected. ^9^^,^^10^ Ritonavir/lopinavir is a combination that is usually used against HIV. ^10^ Lopinavir inhibits some enzymes involved in the virus multiplication cycle, while ritonavir acts as a protector of lopinavir because it degrades very quickly. ^10^ The use of hydroxychloroquine against the new coronavirus has been very controversial. The Spanish Agency for Medicines and Health Products (Aemps) warns that this medicine “has been shown to be effective against SARS-CoV-2 in in vitro studies, but there is still no solid scientific evidence on its efficacy against Covid-19 in humans”. ^11^ Finally, Dexamethasone is a corticosteroid that is emerging as an option for the most serious cases of COVID-19, since it could reduce mortality. ^12^

These compounds are combined with other anti-inflammatory or virus-inhibiting substances, as well as with antibiotics (to treat or prevent secondary bacterial infections) and cytokine inhibitors. ^13^ As for the treatment for infections caused by cold coronaviruses, cases are usually mild and are overcome by following the same steps as with a common cold, ^13^^,^^14^ a new alternative to the treatment for the COVID-19 is needed to improve therapeutic alternatives for this disease that has claimed the lives of multiple people around the world. A series of compounds used, tested, and associated as treatments for SARS-CoV-2 have been selected for this study. These compounds are: Darunavir (Daru), Dexamethasona (Dexame), Dolutegravir (Dolu), Fosamprenavir (Fosam), Ganciclovir (Gan), Insoine (Inso), Lopinavir (Lop), Ritonavir (Rito) and Tipranavir (Tipra). They are related to molecular treatment against SARS-CoV-2 in vitro studies, have been analysed in this study using theoretical techniques such as molecular docking, molecular quantum similarity (MQS) and chemical reactivity descriptors within the Density Functional Theory (DFT).

In our previous publication, ^15^ ligands Ascorbic acid Vitamin C (Asco), Azithromycin (Azythr), Cholecalciferol Vitamin D (Chole) and Hydroxychoroquine (Hidrox); other less known ones such as Abacavir, Acyclovir (Acyc), Amprenavir (Ampre), Baloxavir (Balox), Boceprevir (Boce), Cidofovir (Cido), Edoxudine (Edox) and Emtricitabine (Emitri) were used considering their association for the treatment of SARS-CoV-2. However, in the present study (the second part of an overarching study), a set of entirely different ligands, taking into account the molecular diversity from structural and electronic point of view, have been used to extrapolate and obtain new insights for the SARS-CoV-2 treatment today. The difference between the previous set of ligands ^15^ and the set reported here, is the molecular quantum similarity. The set studied in this work has greater electronic quantum similarity than those presented in Ref. 15, and thus warrants a separate study to specifically investigate the associated impacts of quantum similarity on the results.

## Methods

### System preparation

To conduct the docking analysis, the receptor structures discussed in open access References 9– 14 for the docking experiment were extricated utilizing the following protocols through the crystal Structure of SARS-CoV-2 RNA-dependent RNA polymerase, using Protein Data Bank ( 6M71), see also *Underlying data*, which was adjusted utilizing the protein preparation wizard module of the openly available input file for Schrödinger suite 2017-1. The system preparation has been implemented followed these steps:

i) A key factor on the docking results is the hydrogen bond. For these reasons the hydrogen bond (H-bond) network was optimized, and the protein structure was refined, at physiological pH. This weight was optimized based on the premise that high-resolution structures accurately reflect hydrogen bonding in proteins.
ii) The charge of the ligands on the active site is crucial on the stabilization in the active site. Taking this into account, the protonation states were determined using PropKa utility, part of the Schrödinger suite. This program reaffirms the ionic character of compounds and predicts the pKa values of ionizable groups in proteins and protein-ligand complexes based in the 3D structure.
iii) The possible correlation effects in the heavy atoms were corrected using the Impact Refinement (Impref) module to execute a restrained molecular minimization with heavy atoms constrained to a low root-mean-square deviation (RMSD) from the initial coordinates. ^16^^–^^18^ It is helpful to arrange the observations in serial order of the independent variable when one of the two variables is clearly identifiable as independent.

The influence of pH (acidity or alkalinity) on the SARS-CoV-2 virus, which causes COVID-19, is an interesting topic. However, it’s important to note that SARS-CoV-2 primarily infects and spreads among human cells, and the virus itself doesn’t exist independently in the environment for long periods. The pH level becomes more relevant when considering how the virus interacts with the human body.

### Molecular docking

In our research group the calculation (docking results) were carried out through the freely available Schrödinger suite using the Glide, ^19^^,^^20^ Glide is the program of the Schrödinger suite and was used to obtain the docking results. This program was used with default parameters (that is, the number of poses written per ligand was set to 10,000, and the scaling factors of the vdW radii and the partial atomic charge cut-off were set to the default values 0.80 and 0.15, respectively) and Standard Precision (SP) model has been used for docking outcomes. One of the most important parameters about the docking analysis is the grid. The grid generation implementation has been benchmarked using the target protein of SARS-CoV-2 RNA-dependent RNA polymerase. All molecular Docking has been supported by a molecular dynamic of 30 ns.

### Quantum similarity analysis

*Molecular quantum similarity measure*

An important feature associated with structural and electronic point of view is the Molecular Quantum Similarity Measure (MQSM). The MQSM of the systems A and B, known as Z _AB_, is obtained using the Density Functions (DFs) using Eq. (1).

$$Z_{\mathrm{AB}}=\left\langle \rho_{A}|\Omega |\rho_{B}\right\rangle =\iint \rho_{A}\left(r_{1}\right)\Omega \left(r_{1},r_{2}\right)\rho_{B}\left(r_{2}\right){\mathrm{dr}}_{1}{\mathrm{dr}}_{2}$$
(1)

Studying the nature of the operator Ω( *r*_1_, *r*_2_) with electronic densities for A and B, ^21^^–^^26^ used in Equation 1, provides the information being compared between the two systems while simultaneously designating our measure of similarity. For instance, if the chosen operator is the Dirac delta function (an efficient approach for functions with high peak values, such as the electronic density), i.e., Ω( *r*_1_, *r*_2_) = δ( *r*_1_ - *r*_2_). ^26^^–^^33^ Another widely used alternative is the Coulomb operator, i.e., Ω( *r*_1_, *r*_2_) = | *r*‌ _1_ - *r*_2_|‌‌ ^-1^, resulting in a Coulombic MQSM. ^34^^–^^36^

### Chemical reactivity outcomes

Previously, several investigations have shown the relationship between quantum similarity and chemical reactivity descriptors. ^37^^–^^47^ In addition, the quantum similarity and DFT uses the density function as an object of study of the similarity indexes; specifically, the Coulomb index can be related to electronic factors associated with chemical reactivity.

Using the Frontier Molecular Orbitals (FMO) and the energy gap, the global reactivity indices, such as chemical potential ( *μ*), ^48^ hardness ( *ɳ*), ^49^ and electrophilicity ( *ω*), ^48^^,^^49^ will be calculated.

These chemical reactivity indices ( Eqs. 2- 4) give an idea about the stability of the systems. The chemical potential measures the inclination of electrons to leave the equilibrium system, ^48^ whereas chemical hardness measures the resistance of a chemical species to change its electronic configuration. ^39^

$$\mu \approx \frac{E_{\text{LUMO}}+E_{\text{HOMO}}}{2}$$
(2)

$$\eta \approx E_{\text{LUMO}}-E_{\text{HOMO}}$$
(3)

Electrophilicity index ( *ω*) measure the stabilization energy of the system when it is saturated by electrons from the external environment and is mathematically defined as ^48^^,^^49^:

$$\omega =\frac{\mu^{2}}{2\eta }$$
(4)

In this work, the local reactivity descriptors are the Fukui functions. Equations (5, 6) represents the response of the chemical potential of a system to changes in the external potential.

$$f^{+}(\vec{r})\approx {\left|\text{LUMO}(\vec{r})\right|}^{2}$$
(5)

$$f^{-}(\vec{r})\approx {\left|\text{HOMO}(\vec{r})\right|}^{2}$$
(6)

Where $\left(f^{+}(\vec{r})\right)$ is for nucleophilic attack and $\left(f^{-}(\vec{r})\right)$ for the electrophilic attack. ^50^^–^^54^

All the calculations were carried out using the method B3LYP. B3LYP is one of the most used Density-Functional Theory (DFT) approaches. It is capable of predicting molecular structures and other properties according to the experimental data ^55^ and the basis set 6-311G (d,p) ^56^ which is the result of adding a correction to the 6-311G(d) basis set leading to calculations of electronegativity, hardness, reactivity indices and frontier molecular orbitals with a similar quality to those obtained with much larger basis sets (such as Aug-cc-pVQZ and Aug-cc-pV5Z). This method/basis set has been used in combination with Gaussian 16 package ^57^ and GaussView, Version 6.1 ^58^ a free, alternative software that carries out a similar function is ORCA 5.0.3.

## Results and discussion

### Molecular analysis

One of the objectives of this project involves an analysis about molecular coupling of the compounds on the active site. For this reason, a study about the best conformations on the active site of the selected compounds was carried out. Please note that all files associated with the results are available in *Underlying data.* ^59^

**Figure 1.**
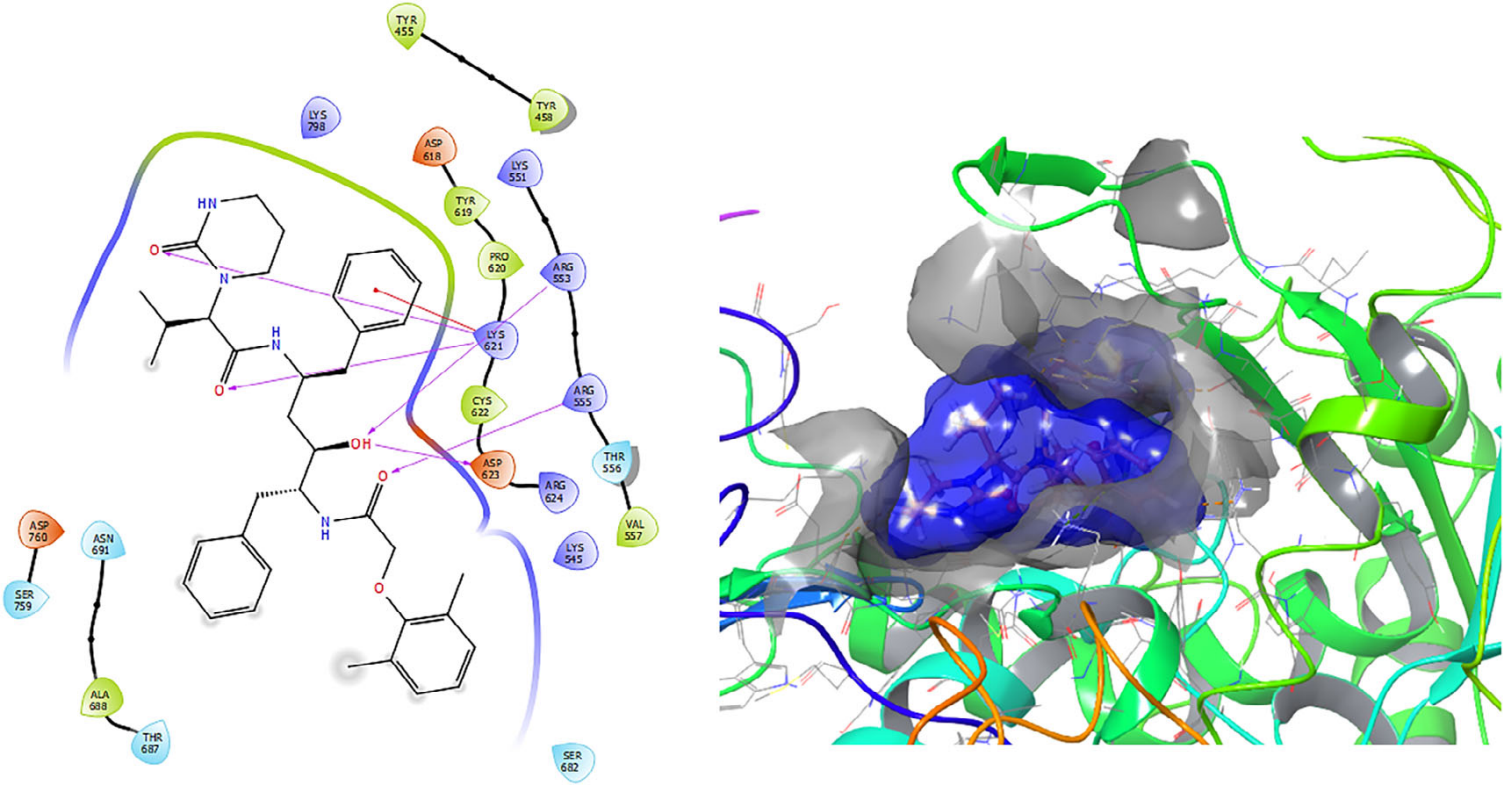
Molecular docking results for the Lopinavir using Schrödinger suite 2017-1.

Figure 1 shows the conformation with the highest RMSD, in this conformation Lopinavir has a π-π stacking interaction with residue LYS621 with a length of 1.56Å. On the other hand, with this same residue they have an H-bond with a length of 1.62Å and 1.72Å, respectively. Additionally, this compound has an H-bond with residues ARG553, ARG555 and ASP623 with lengths of 1.56Å, 1.64Å and 1.42Å, respectively.

**Figure 2.**
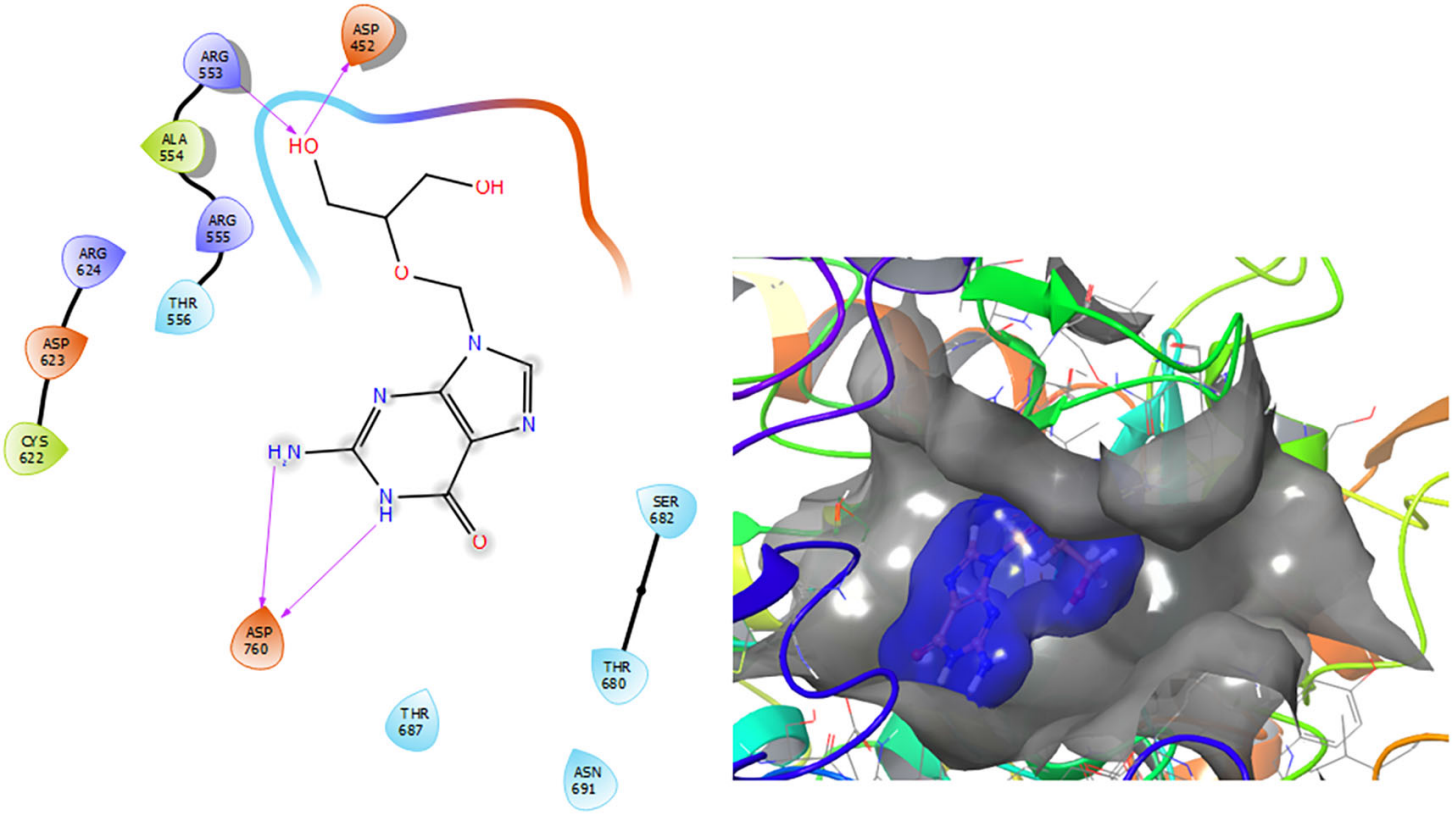
Molecular docking results for the Ganciclovir using Schrödinger suite 2017-1.

Figure 2 shows that the Ganciclovir compound has a H-Bond with the residue ARG553 with a length of 1.58Å and ASP452 with a length of 1.62Å. Also, this compound has two H-bonds with the residue ASP760 with lengths of 1.59Å and 1.63Å, respectively.

**Figure 3.**
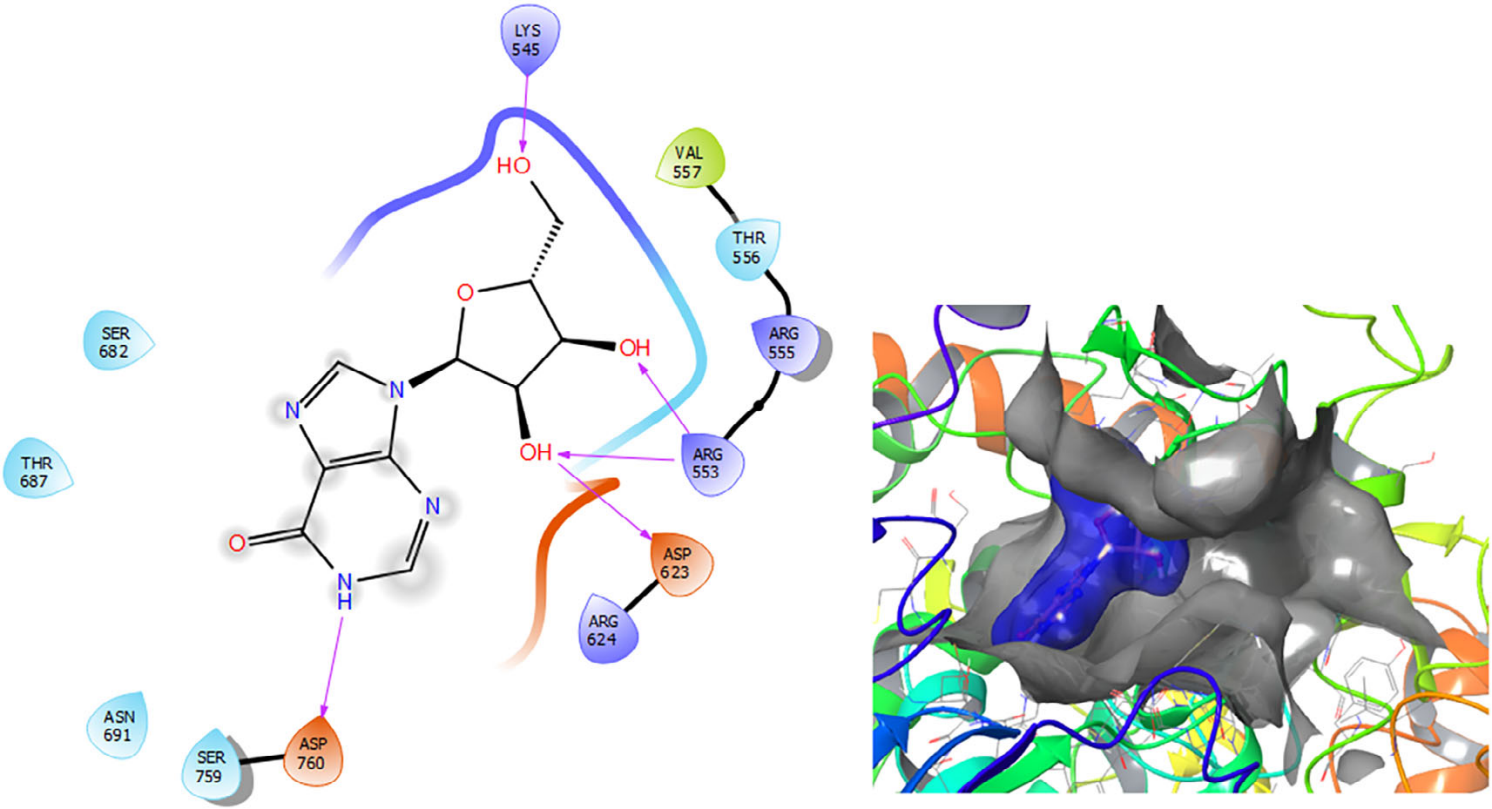
Molecular docking results for the Insoine using Schrödinger suite 2017-1.

Figure 3 shows that the Insoine compound has a H-bond with the residue ASP760 with a length of 1.65Å, with the residue ARG553 have two H-bonds with lengths of 1.58Å and 1.61Å, respectively. Finally, it compounds have a H-bond with a length of 1.48Å.

**Figure 4.**
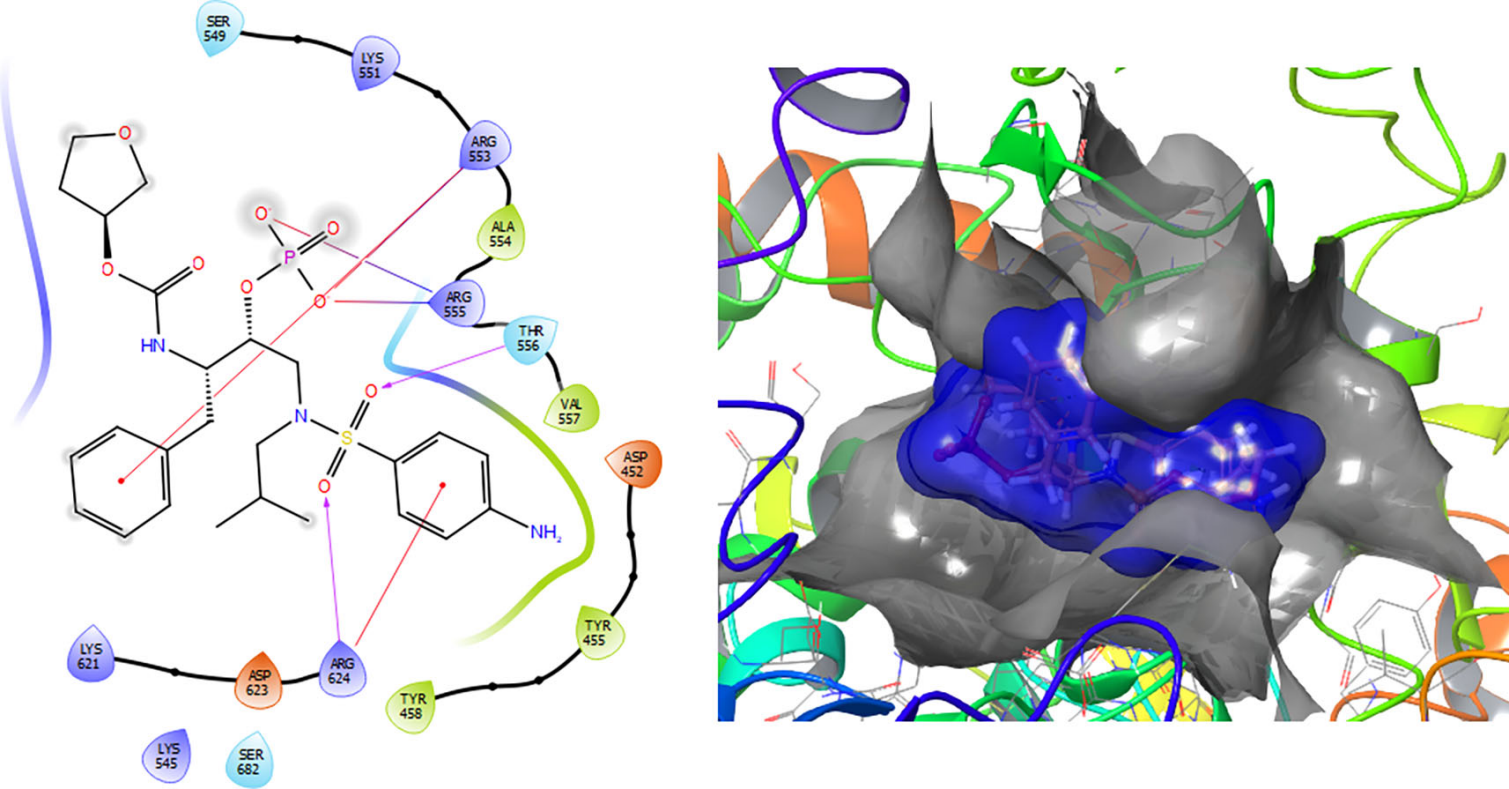
Molecular docking results for the Fosamprenavir using Schrödinger suite 2017-1.

The Fosamprenavir compound has two a π–π stacking interactions with the residue ARG624 with a length of 1.62Å, another interaction with the residue ARG553 with a length of 1.82Å (see Figure 4). Also have a H-bond with a residue ARG553 with a length of 1.62Å, another H-bond with ARG624 with a length of 1.57Å and with the residue THR556 with a length of 1.61Å.

**Figure 5.**
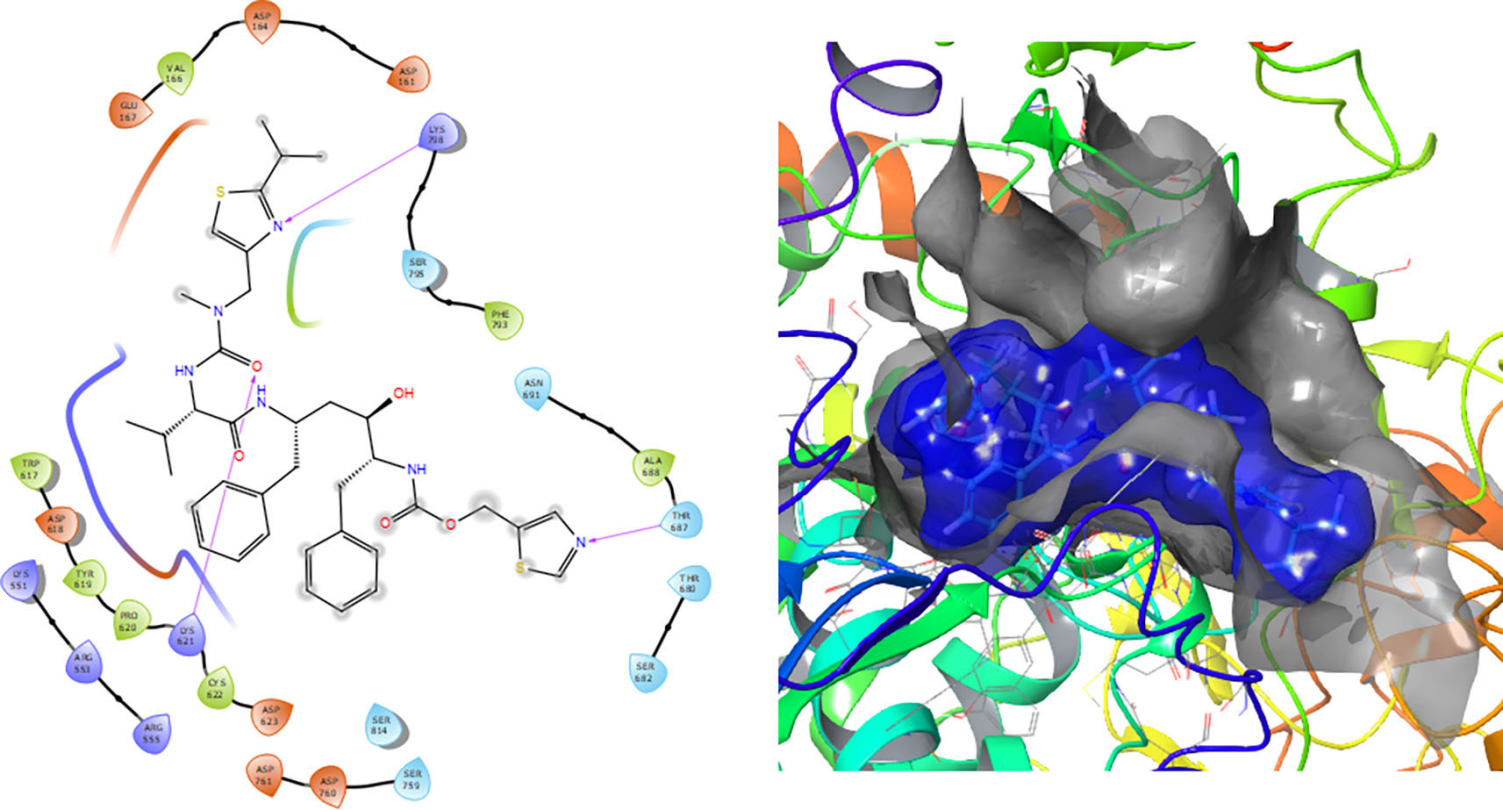
Molecular docking results for the Ritonavir using Schrödinger suite 2017-1.

Ritonavir has a -H bond with the residues LYS298 and LYS621 with a length of 1.26Å and 1.43Å ( Figure 5). Also has a H-bond with the residue THR687 with a length 1.13 Å, respectively.

**Figure 6.**
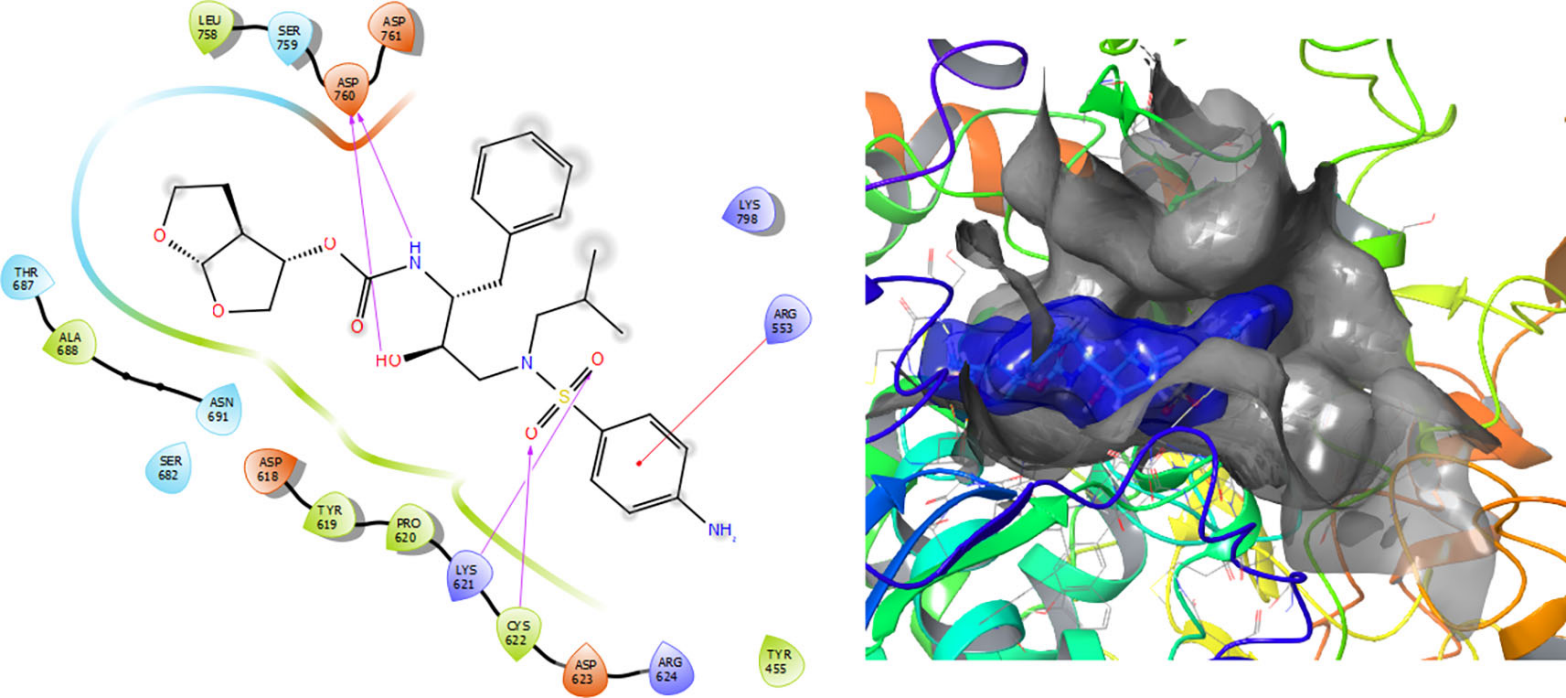
Molecular docking results for the Darunavir using Schrödinger suite 2017-1.

Darunavir compound has a π–π stacking interactions with the residue ARG553 with a length of 1.41Å (as can be seen in Figure 6). Also have a H-bond with the residue CYS622 with a length 1.38Å, with the residue LYS621 with a length of 1.68Å and two H-bonds with lengths of 1.48Å and 1.38Å, respectively.

**Figure 7.**
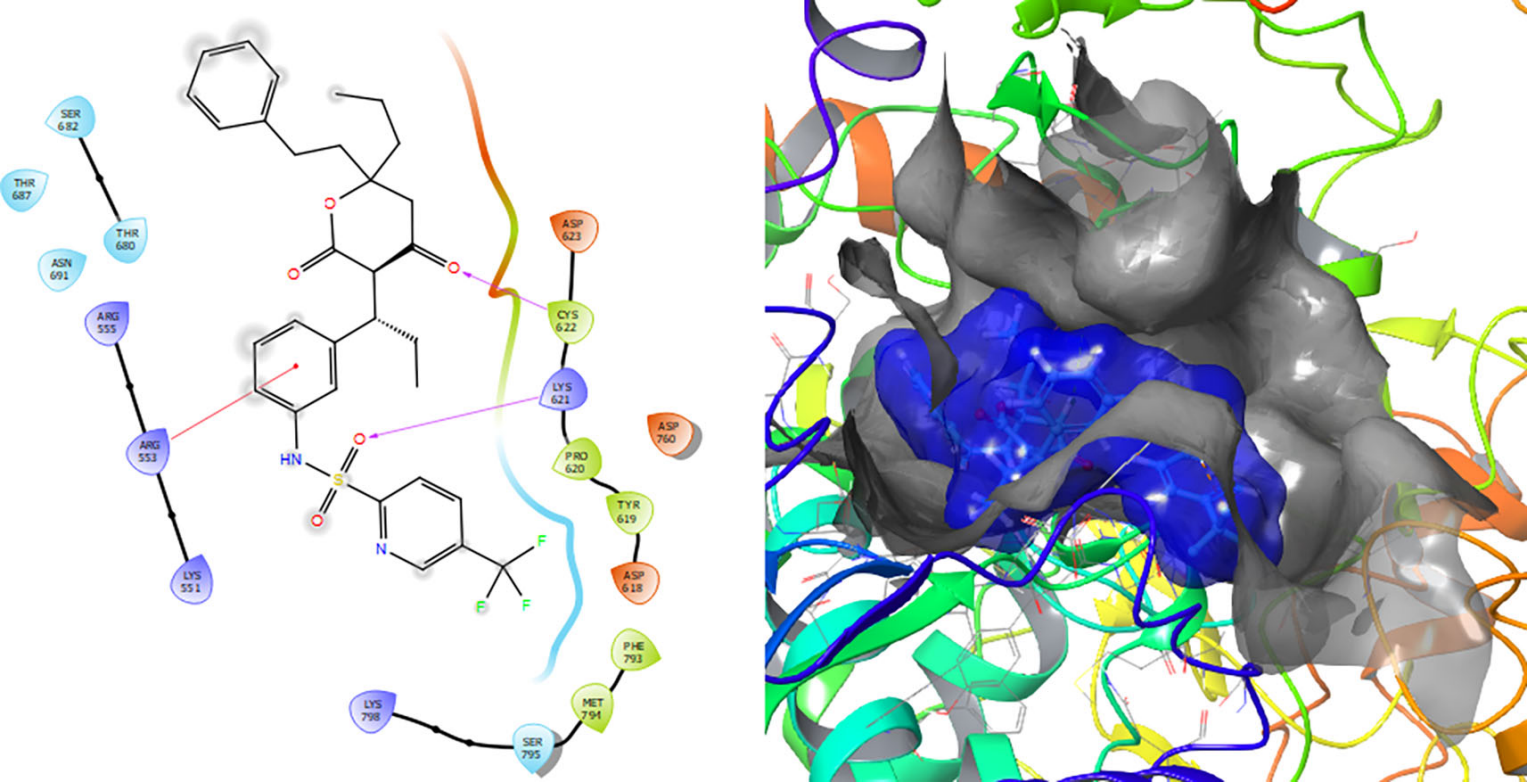
Molecular docking results for the Tipranavir using Schrödinger suite 2017-1.

Figure 7 shows that the Tipranavir compound has a π–π stacking interactions with three H-bonds with the residue CYS622 with a length of 1.66Å, with a residue LYS621 with a length of 1.63Å and a π–π stacking interactions with length of 1.46Å.

**Figure 8.**
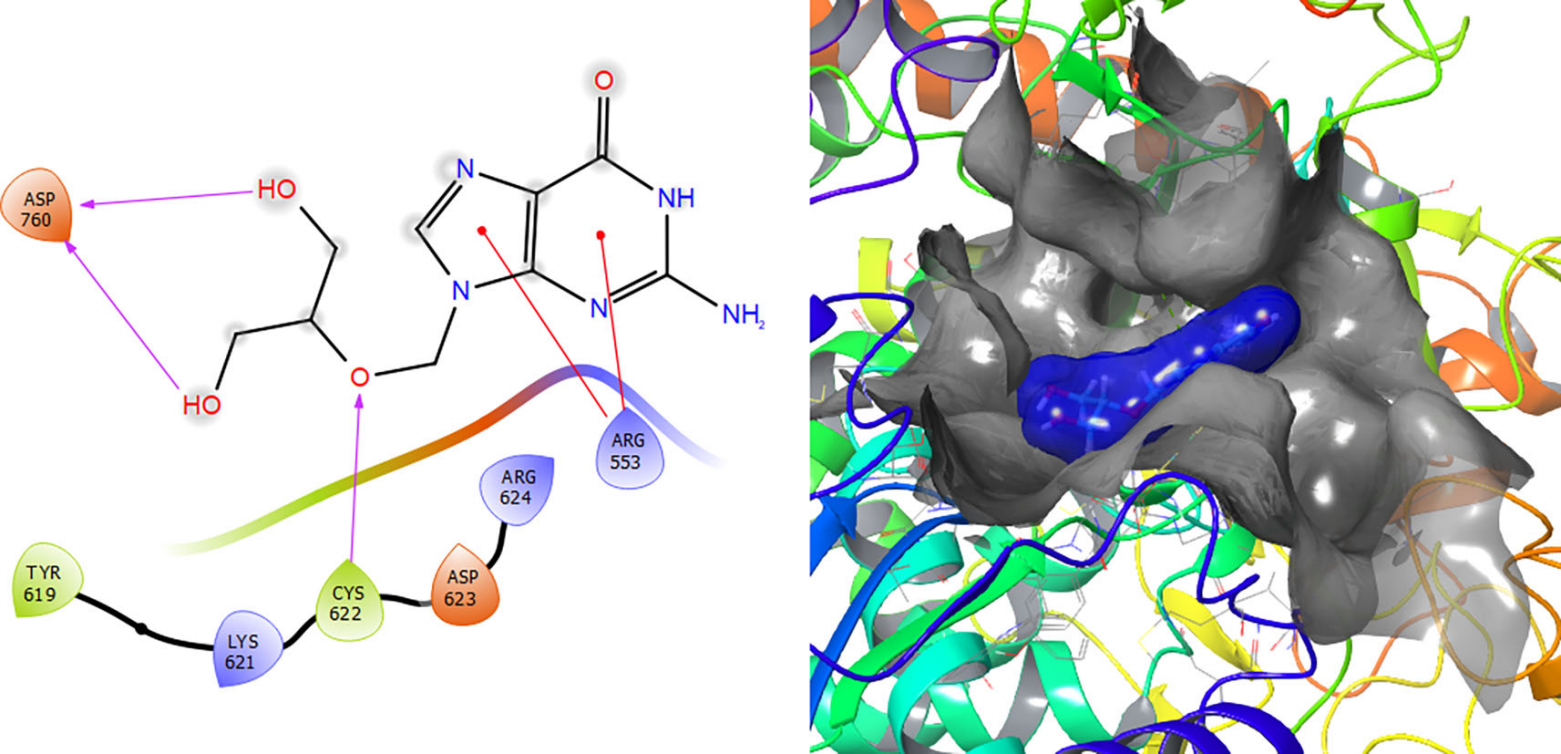
Molecular docking results for the Ganciclovir using Schrödinger suite 2017-1.

Ganciclovir compound has two π–π stacking interactions with the residue ARG553 with lengths of 1.52Å and 1.61Å (see Figure 8). On the other hand, it compounds have a H-bond with the residue CYS622 with a length of 1.58Å and two H-bonds with the residue ASP760 with lengths of 1.61Å and 1.71Å, respectively.

**Figure 9.**
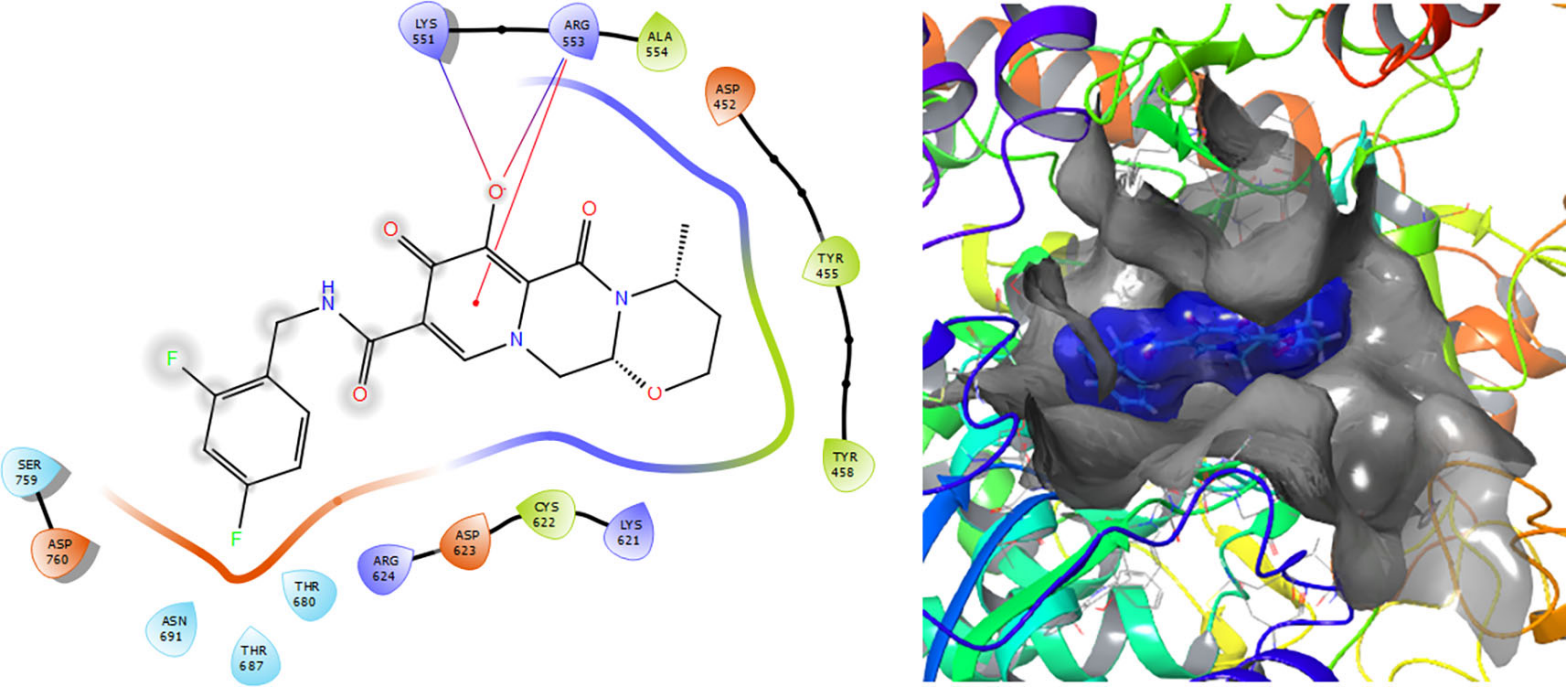
Molecular docking results for the Dolutegravir using Schrödinger suite 2017-1.

The Molecular docking results for Dolutegravir (see Figure 9) involves a π–π stacking interactions with lengths of 1.64Å and two H-bonds with the residues LYS551 and ARG553 with lengths of 1.57Å and 1.59Å, respectively.

### Molecular quantum similarity

In the Table 1 we can see the structural similarity results for the molecular reaction set. This analysis was developed with the aim the find the common features along the reaction set. From structural point of view the Darunavir (Daru), Dexamethasona (Dexame), Dolutegravir (Dolu), Fosamprenavir (Fosam), Ganciclovir (Gan), Insoine (Inso), Lopinavir (Lop), Ritonavir (Rito) and Tipranavir (Tipra) were analysed, the highest overlap similarity is between the compounds Fosam and Daru (0.343) with a Euclidean distance of 6.946 (see Table 2); Lop vs Fosam (0.414) with a Euclidean distance of 6.694 and Inso vs Gan (0.510) with a Euclidean distance of 0.510. These low values obtained are related with steric effects between structures. Additionally, they do not have a skeleton in common and substitutes groups have hight differences.

**Table 1.** Molecular quantum similarity indices using overlap descriptors ( Equation 6).

O_Hab	Daru	Dexame	Dolu	Fosam	Gan	Inso	Lop	Rito	Tipra
**Daru**	1.000								
**Dexame**	0.247	1.000							
**Dolu**	0.191	0.271	1.000						
**Fosam**	0.343	0.280	0.199	1.000					
**Gan**	0.298	0.324	0.254	0.286	1.000				
**Inso**	0.248	0.239	0.317	0.248	0.510	1.000			
**Lop**	0.322	0.314	0.179	0.414	0.367	0.192	1.000		
**Rito**	0.276	0.336	0.261	0.243	0.257	0.178	0.293	1.000	
**Tipra**	0.242	0.212	0.271	0.234	0.264	0.237	0.149	0.229	1.000

**Table 2.** Molecular quantum similarity indices using Euclidean distances and overlap descriptor (Equation 11).

O_Dab	Daru	Dexame	Dolu	Fosam	Gan	Inso	Lop	Rito	Tipra
**Daru**	0.000								
**Dexame**	6.855	0.000							
**Dolu**	7.347	6.533	0.000						
**Fosam**	6.946	6.871	7.477	0.000					
**Gan**	5.905	5.216	5.786	6.153	0.000				
**Inso**	6.503	6.009	5.979	6.686	4.039	0.000			
**Lop**	7.059	6.713	7.565	6.694	5.866	6.914	0.000		
**Rito**	7.454	6.779	7.354	7.765	6.468	7.168	7.504	0.000	
**Tipra**	7.625	7.367	7.301	7.813	6.445	6.925	8.233	7.992	0.000

Due to the low indices of structural similarity, the electronic similarity has been calculated (see Table 2). These values highest values for electronic similarity are Fosam vs Dexame (0.884) with a Euclidean distance of 36.295; Rito vs Dexame (0.896) with a Euclidean distance of 43.347 (see Table 4), Lop vs Fosam (0.915) with a Euclidean distance of 33.223; and Rito vs Lop (0.881) with a Euclidean distance of 41.726. Unlike the values of structural similarity, the values of electronic similarity are above of 0.5. These values are supported with the Euclidean distances. We think that the structures, despite being structurally different, are electronically very similar.

### Reactivity analysis and Fukui function comparison

Since the electronic similarity indices (see Tables 3 and 4) are higher than the structural ones, this section deepens on the electronic effects associated with the highest values of electronic similarity. In the previous section we have studied which ligands have greater structural and electronic similarity. To continue this analysis, from the point of view of chemical reactivity, several ligand pairs have been selected from those with indices indicating greater electronic and structural similarity. Table 5 shows the global parameters chemical potential, chemical hardness, lobal S softness and global electrophilicity that have been calculated in order to compare the chemical reactivity of these ligands, analysing the values of this table it can be concluded that Insoine, Ganciclovir and Fosamprenavir have a set of parameters that are closer to each other. Since the analysis of the global parameters is limited, we will complete it with the comparison of some local descriptor functions. The electrophile and nucleophile Fukui functions (as a measure of reactivity) are then compared using the Frontier Molecular Orbital (FMO) approach. The electrophilic-nucleophilic character of the following functions also shows those molecular areas that are most likely to form charge-donating interactions (basically by charge delocalisation). These types of interactions are important and are difficult to determine using docking analysis.

**Table 3.** Molecular quantum similarity indices using Coulomb descriptor (Equation 7).

C_Hab	Daru	Dexame	Dolu	Fosam	Gan	Inso	Lop	Rito	Tipra
**Daru**	1.000								
**Dexame**	0.796	1.000							
**Dolu**	0.746	0.736	1.000						
**Fosam**	0.831	0.884	0.734	1.000					
**Gan**	0.588	0.812	0.678	0.622	1.000				
**Inso**	0.642	0.796	0.723	0.562	0.861	1.000			
**Lop**	0.876	0.829	0.750	0.915	0.808	0.732	1.000		
**Rito**	0.820	0.896	0.787	0.816	0.688	0.667	0.881	1.000	
**Tipra**	0.794	0.747	0.765	0.853	0.736	0.717	0.616	0.763	1.000

**Table 4.** Molecular quantum similarity indices using Euclidean distance and Coulomb descriptor (Equation 11).

C_Dab	Daru	Dexame	Dolu	Fosam	Gan	Inso	Lop	Rito	Tipra
**Daru**	0.000								
**Dexame**	43.566	0.000							
**Dolu**	48.063	42.659	0.000						
**Fosam**	43.088	36.295	51.575	0.000					
**Gan**	58.487	36.505	42.733	60.307	0.000				
**Inso**	55.233	36.121	40.086	62.719	21.878	0.000			
**Lop**	39.673	46.948	54.617	33.223	57.091	58.720	0.000		
**Rito**	50.153	43.347	55.115	50.812	67.829	67.030	41.726	0.000	
**Tipra**	48.143	51.301	49.703	41.523	56.037	55.175	70.071	57.583	0.000

**Table 5.** Global chemical reactivity indices (in eV) for some selected ligands.

Compounds	Chemical Potential ( *μ*), eV	Chemical Hardness ( *ɳ*), eV	Softness ( *S*), eV	Electrophilicity ( *ω*), eV
Insoine	-3.92	5.22	0.192	1.47
Ganciclovir	-3.51	5.24	0.191	1.18
Lopinavir	-3.57	5.90	0.169	1.08
Fosamprenavir	-3.99	5.24	0.191	1.52
Darunavir	-3.68	5.28	0.189	1.28
Dexamethasone	-4.38	4.87	0.205	1.97
Ritonavir	-3.66	5.46	0.183	1.23

**Figure 10.**
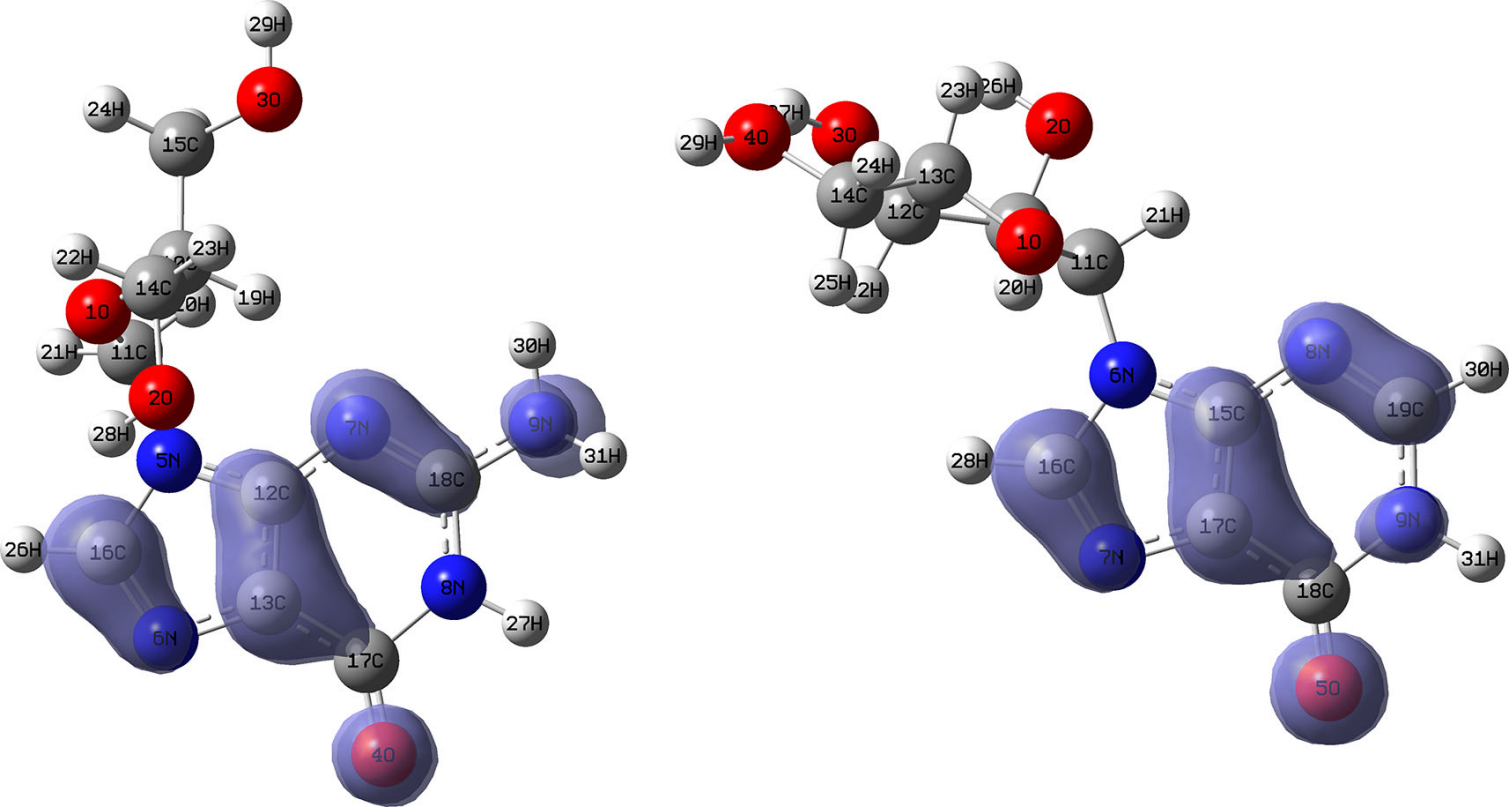
Fukui function $f^{-}(\vec{r})$ calculated under the Frontier Molecular Orbital (FMO) approximation $\left({\left|\mathrm{HOMO}(\vec{r})\right|}^{2}\right)$ for the ligands Insoine (left) and Ganciclovir (right). Isovalue: 0.002 in both cases. Figure created using Schrödinger suite 2017-1.

**Figure 11.**
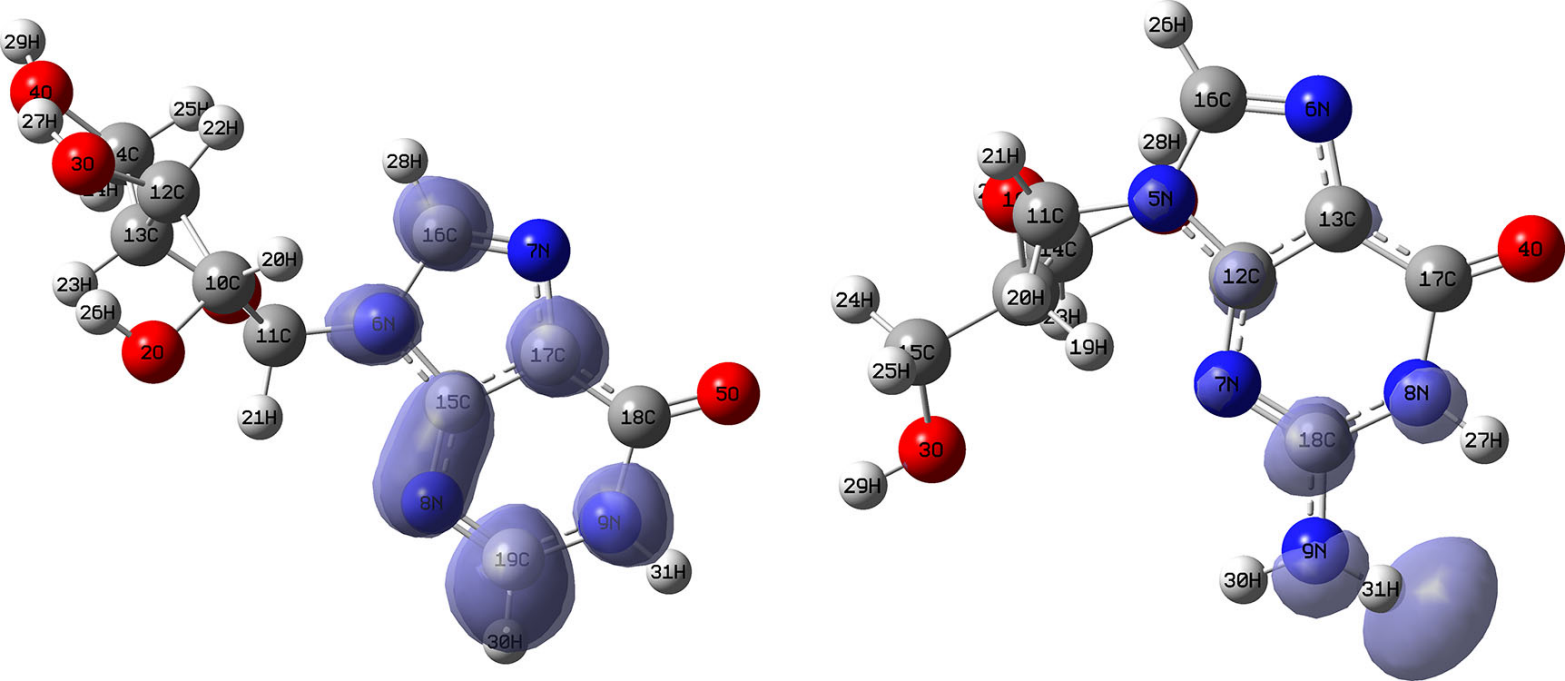
Fukui function $f^{+}(\vec{r})$ calculated under the Frontier Molecular Orbital (FMO) approximation $\left({\left|\mathrm{LUMO}(\vec{r})\right|}^{2}\right)$ for the ligands Insoine (left) and Ganciclovir (right). Isovalue: 0.002 in both cases.

Figures 10 and 11 show the Fukui $f^{-}(\vec{r})$ and $f^{+}(\vec{r})$ functions corresponding to the Insoine and Ganciclovir ligands. Figure 10 shows a strong similarity between the two functions, which may indicate that both ligands have a similar nucleophilic behaviour and/or that they have a similar tendency to donate electronic charge. On the other hand, Figure 11 shows significantly different descriptor functions so we would expect different electrophilic behaviour for these ligands and/or a very different tendency in relation to the possible charge-withdrawing interactions. The Fukui Functions maps were obtained using Highest Occupied Molecular Oribital (HOMO) maps and the Lowest Unoccupied Molecular Orbital (LUMO) maps).

**Figure 12.**
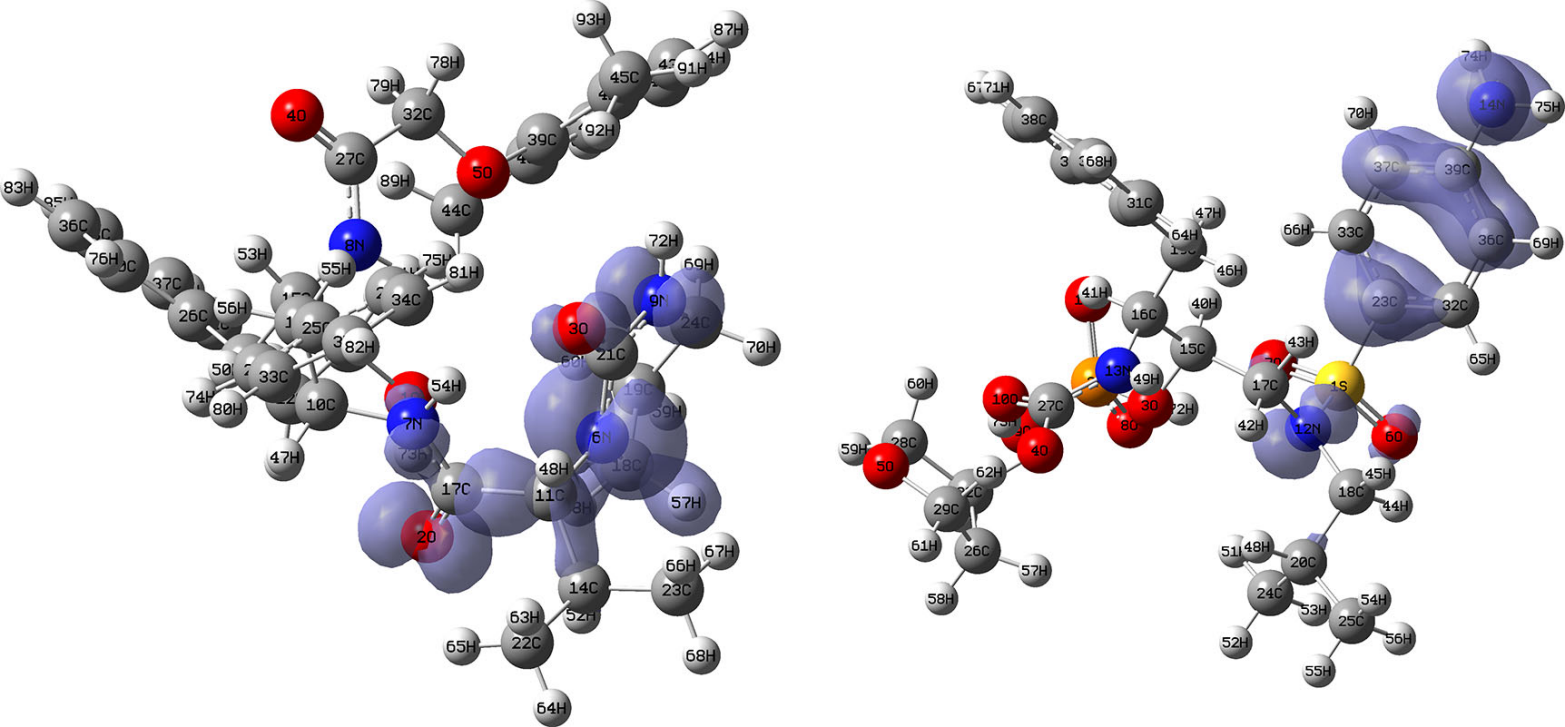
Fukui function $f^{-}(\vec{r})$ calculated under the Frontier Molecular Orbital (FMO) approximation $\left({\left|\mathrm{HOMO}(\vec{r})\right|}^{2}\right)$ for the ligands Lopinavir (left) and Fosamprenavir (right). Isovalue: 0.002 in both cases. Figure created using Schrödinger suite 2017-1.

**Figure 13.**
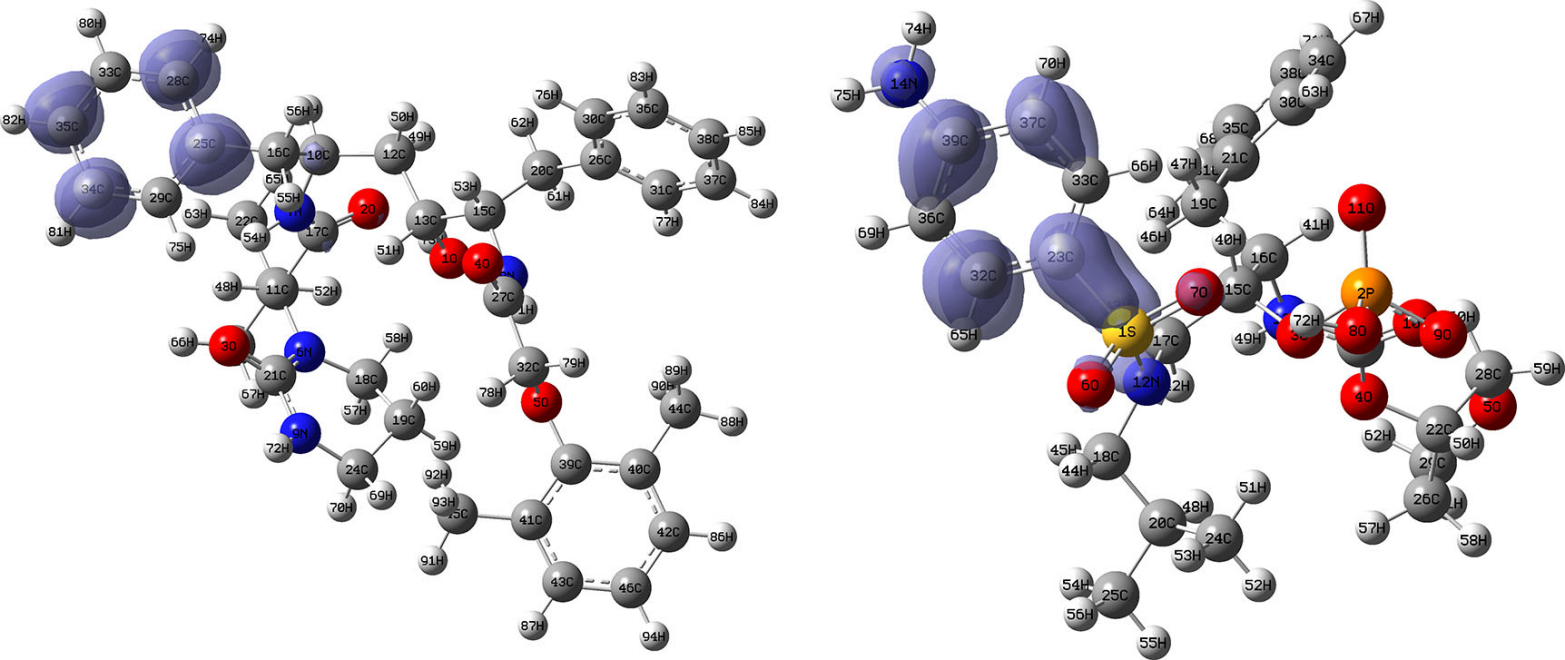
Fukui function $f^{+}(\vec{r})$ calculated under the Frontier Molecular Orbital (FMO) approximation $\left({\left|\mathrm{LUMO}(\vec{r})\right|}^{2}\right)$ for the ligands Lopinavir (left) and Fosamprenavir (right). Isovalue: 0.002 in both cases.

Figures 12 and 13 show the Fukui functions for the ligands Lopinavir and Fosamprenavir. In Figure 12 it can be seen that there is no similarity between the two functions, indicating that the ligands have different nucleophilic behaviour and/or show a different trend in electronic charge donation. On the other hand, Figure 13 shows descriptor functions with a certain resemblance so we would expect comparable electrophilic behaviour between these ligands although the similarity is moderate. Figure created using Schrödinger suite 2017-1.

**Figure 14.**
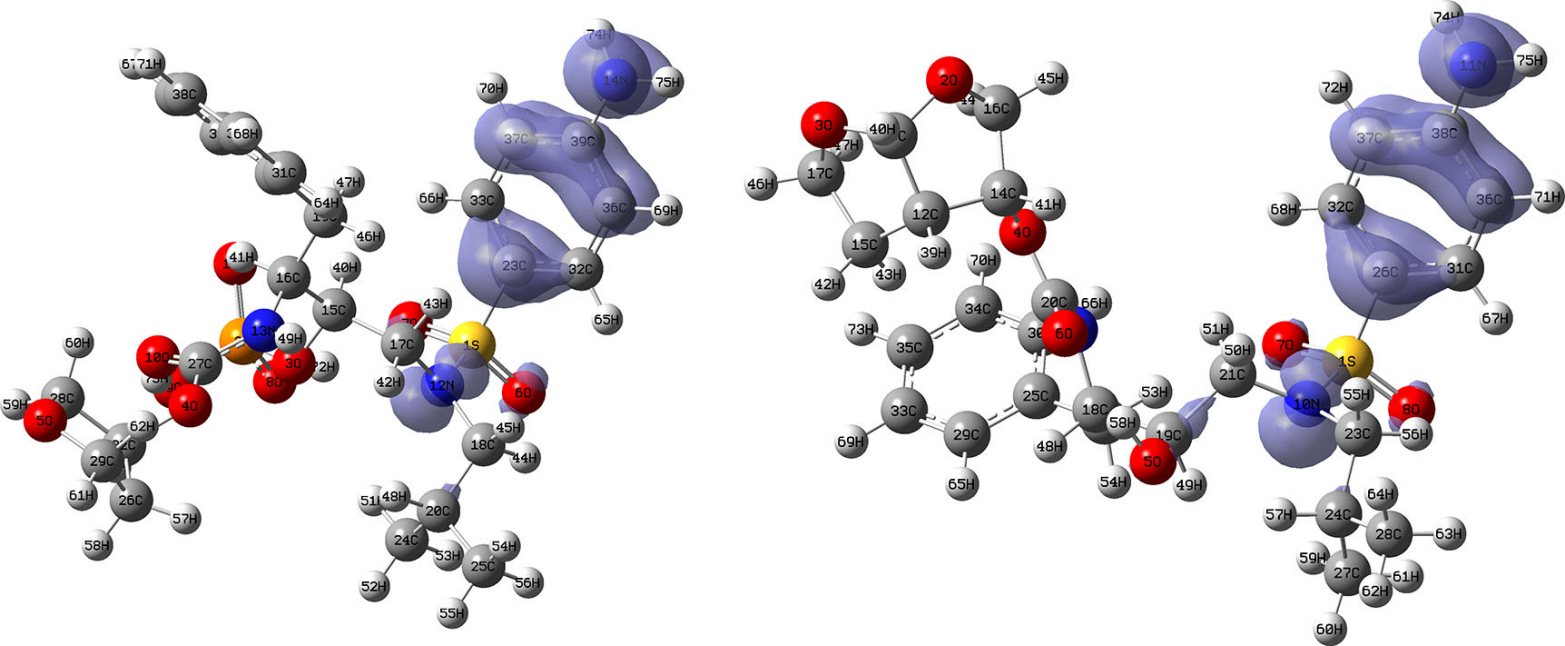
Fukui function $f^{-}(\vec{r})$ calculated under the Frontier Molecular Orbital (FMO) approximation $\left({\left|\mathrm{HOMO}(\vec{r})\right|}^{2}\right)$ for the ligands Fosamprenavir (left) and Darunavir (right). Isovalue: 0.002 in both cases. Figure created using Schrödinger suite 2017-1.

**Figure 15.**
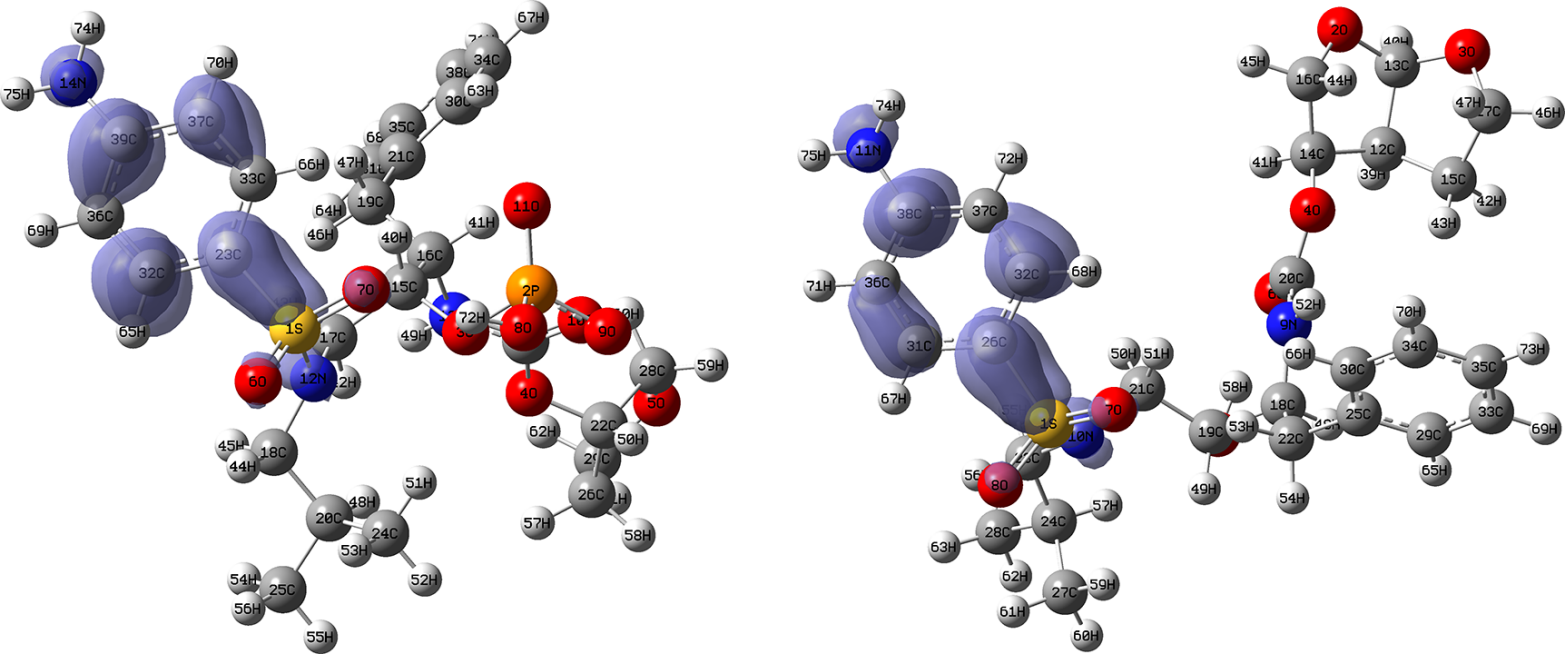
Fukui function $f^{+}(\vec{r})$ calculated under the Frontier Molecular Orbital (FMO) approximation $\left({\left|\mathrm{LUMO}(\vec{r})\right|}^{2}\right)$ for the ligands Fosamprenavir (left) and Darunavir (right). Isovalue: 0.002 in both cases. Figure created using Schrödinger suite 2017-1.

Figures 14 and 15 show the Fukui functions for the ligands Fosamprenavir and Darunavir. Figure 14 shows a strong similarity between the two functions, which may indicate that both ligands have a similar nucleophilic behaviour and/or that they have a similar tendency to donate electronic charge. On the other hand, Figure 15 shows very similar descriptor functions so we would expect comparable electrophilic behaviour between these ligands. Figure created using Schrödinger suite 2017-1.

**Figure 16.**
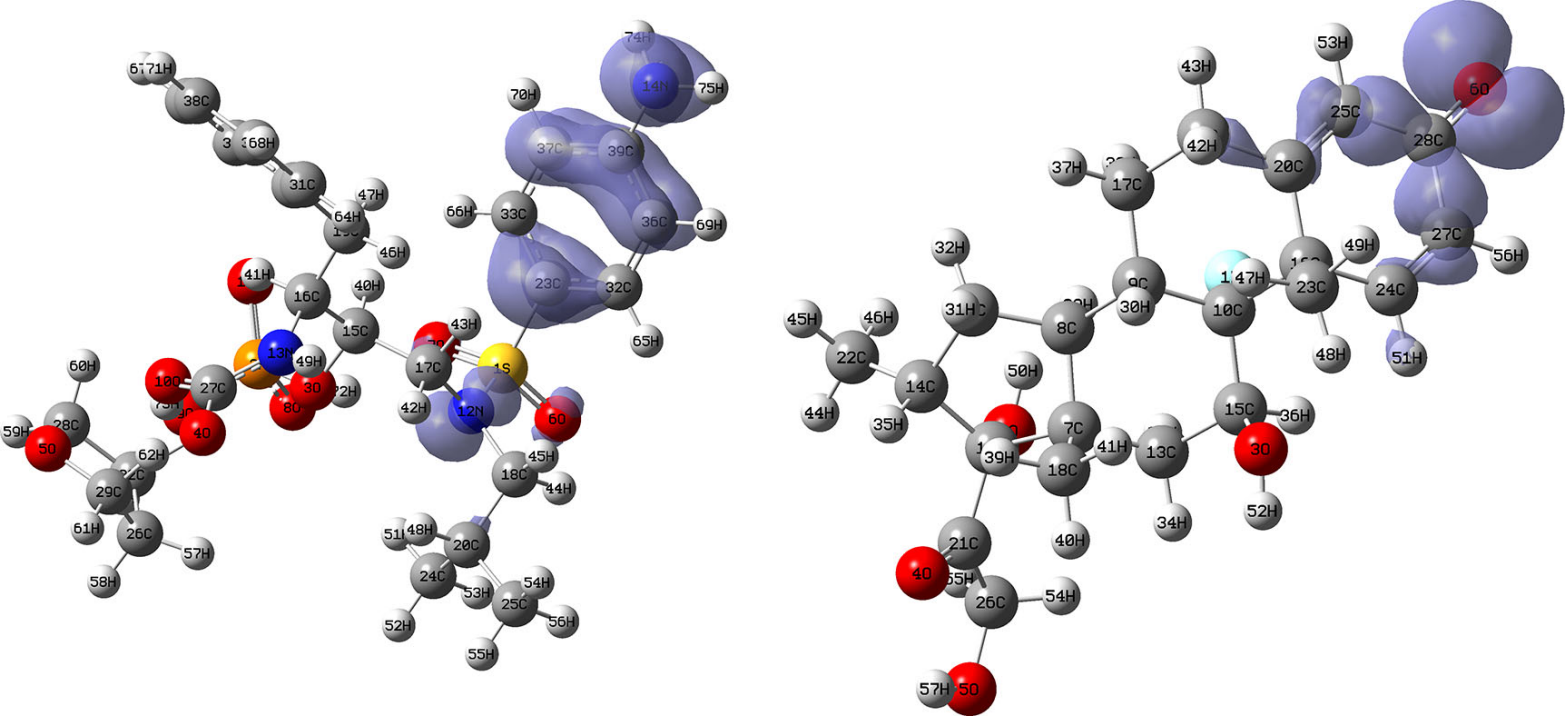
Fukui function $f^{-}(\vec{r})$ calculated under the Frontier Molecular Orbital (FMO) approximation $\left({\left|\mathrm{HOMO}(\vec{r})\right|}^{2}\right)$ for the ligands Fosamprenavir (left) and Dexamethasona (right). Isovalue: 0.002 in both cases. Figure created using Schrödinger suite 2017-1.

**Figure 17.**
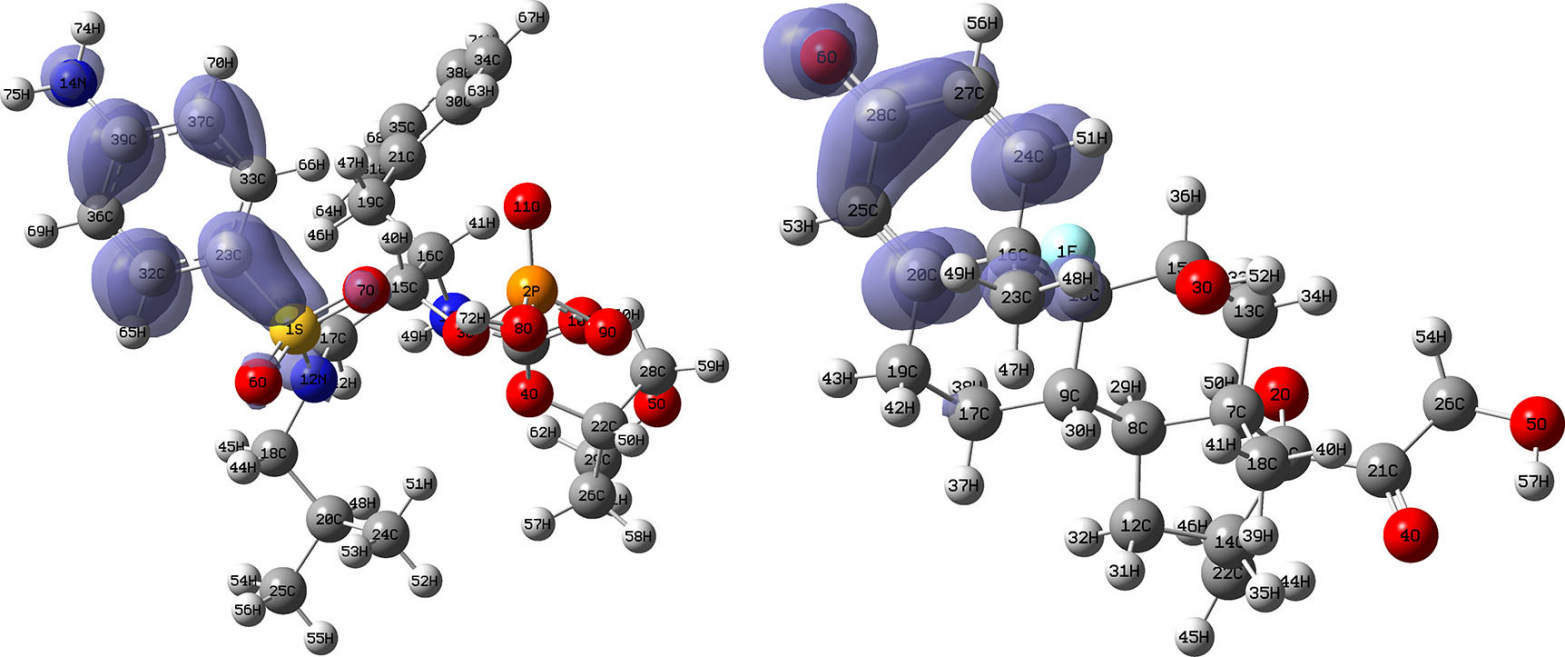
Fukui function $f^{+}(\vec{r})$ calculated under the Frontier Molecular Orbital (FMO) approximation $\left({\left|\mathrm{LUMO}(\vec{r})\right|}^{2}\right)$ for the ligands Fosamprenavir (left) and Dexamethasona (right). Isovalue: 0.002 in both cases. Figure created using Schrödinger suite 2017-1.

Figures 16 and 17 show the Fukui functions for the ligands Fosamprenavir and Dexamethasone. In Figure 16 it can be seen that there is no similarity between the two functions, indicating that the ligands have different nucleophilic behaviour and/or show a different trend in electronic charge donation. On the other hand, Figure 17 shows descriptor functions with a certain resemblance so we would expect comparable electrophilic behaviour between these ligands although the similarity is moderate.

**Figure 18.**
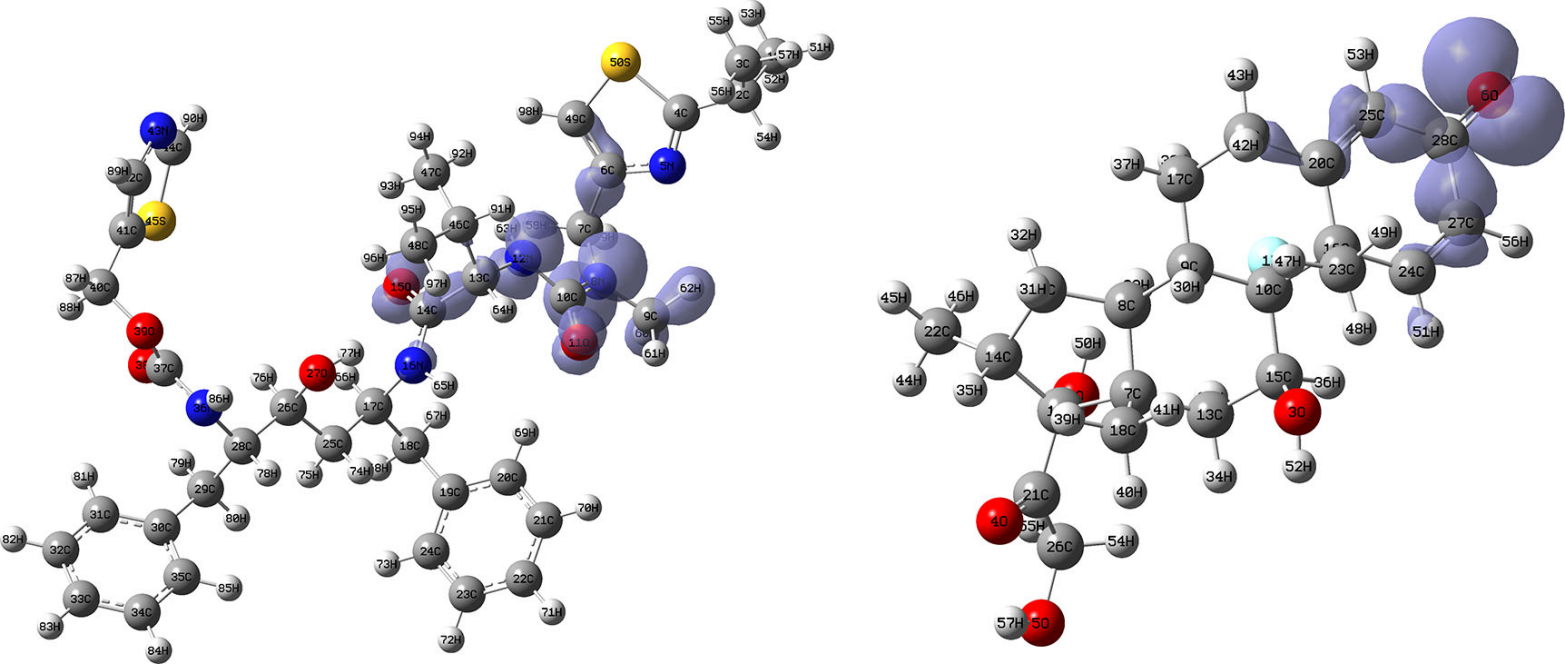
Fukui function $f^{-}(\vec{r})$ calculated under the Frontier Molecular Orbital (FMO) approximation $\left({\left|\mathrm{HOMO}(\vec{r})\right|}^{2}\right)$ for the ligands Ritonavir (left) and Dexamethasona (right). Isovalue: 0.002 in both cases. Figure created using Schrödinger suite 2017-1.

**Figure 19.**
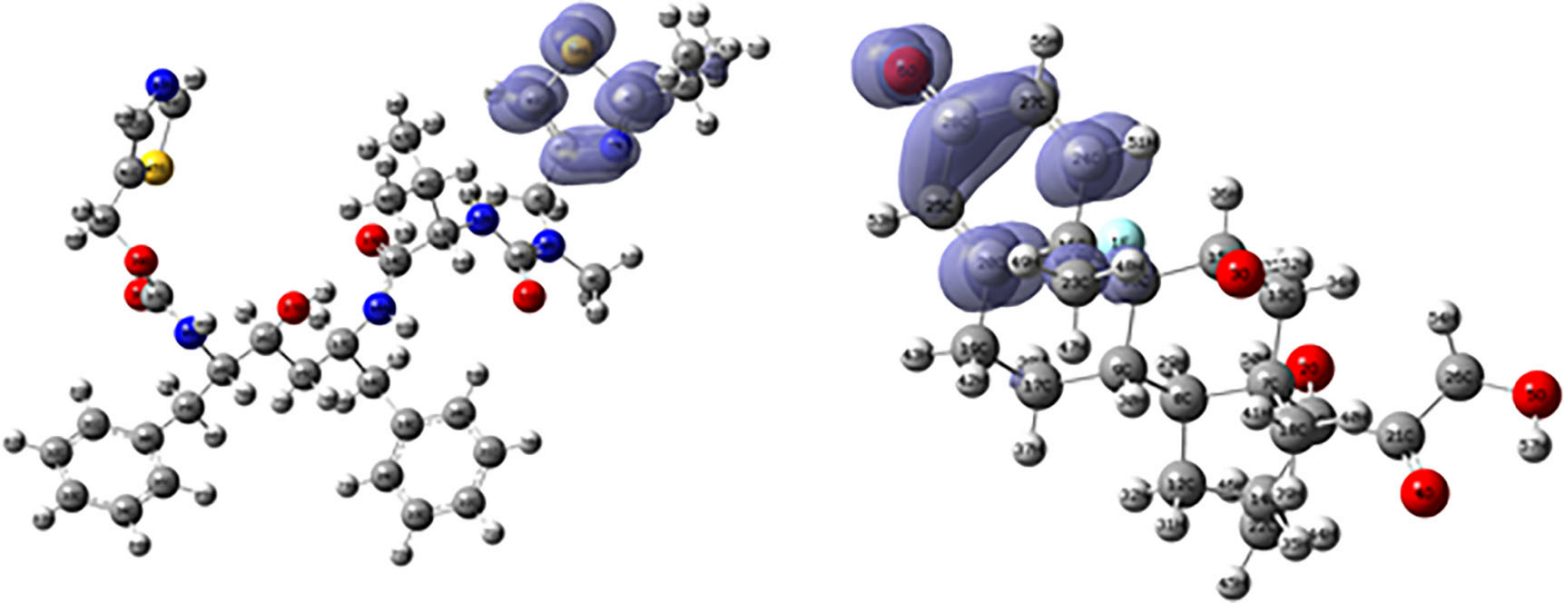
Fukui function $f^{+}(\vec{r})$ calculated under the Frontier Molecular Orbital (FMO) approximation $\left({\left|\mathrm{LUMO}(\vec{r})\right|}^{2}\right)$ for the ligands Ritonavir (left) and Dexamethasona (right). Isovalue: 0.002 in both cases. Figure created using Schrödinger suite 2017-1.

Figures 18 and 19 show the Fukui functions for the ligands Ritonavir and Dexamethasone. In Figure 18 it can be clearly seen that there is no similarity between the two functions, so we think that these ligands have different nucleophilic behaviour. On the other hand, Figure 19 shows descriptor functions with a certain resemblance, so we would expect comparable electrophilic behaviour between these ligands, although the resemblance is very limited.

## Conclusions

In this study, the series of compounds, namely Darunavir (Daru), Dexamethasone (Dexame), Dolutegravir (Dolu), Fosamprenavir (Fosam), Ganciclovir (Gan), Insoine (Inso), Lopinavir (Lop), Ritonavir (Rito) and Tipranavir (Tipra), used against SARS-CoV-2 in vitro studies, have been analysed by molecular docking, molecular quantum similarity and chemical reactivity indices to study their active site stabilisation interactions from a structural and electronic point of view.

From the molecular docking results, it was observed that Lopinavir, Ganciclovir, Insoin, Fosamprenavir, Ritonavir, Tipranavir, Ganciclovir and Dolutegravir show good active site stabilisation with at least one -H bond in each conformation. To further investigate the active site stabilisation of each ligand, a density functional theory (DFT) analysis of quantum similarity and reactivity was developed.

From the structural point of view, the highest overlap similarity is between the compounds Fosam vs Daru (0.343), Lop vs Fosam (0.414) and Inso vs Gan (0.510). From the electronic point of view, the highest Coulomb similarity is between the compounds Fosam vs Dexame (0.884); Rito vs Dexame (0.896), Lop vs Fosam (0.915) and Rito vs Lop (0.881). Finally, with these comparisons, chemical reactivity analysis was performed from a global and local point of view. These Fukui functions were related to charge-donation interactions.

## Data Availability

Protein Data Bank: SARS-CoV-2 RNA-dependent RNA polymerase. Accession number 6M71; https://www.rcsb.org/structure/6M71. Harvard Dataverse: Replication data for Study of a series of ligands used as inhibitors of the SARS-CoV-2 virus. https://doi.org/10.7910/DVN/7KFPUT. ^59^ This project contains the following underlying data:
- Optimized structure of Darunavir (Daru). - Optimized structure of Dexamethasona (Dexame). - Optimized structure of Dolutegravir (Dolu). - Optimized structure of Fosamprenavir (Fosam). - Optimized structure of Ganciclovir (Gan). - Optimized structure of Insoine (Inso). - Optimized structure of Lopinavir (Lop). - Optimized structure of Ritonavir (Rito). - Optimized structure of Tipranavir (Tipra). Each structure has the following file extensions:
- .out (output file of Gaussian 09 calculations). - .chk (output file of the Gaussian 09 calculations used to generate the contour maps). - .gif (input file of the Gaussian 09 calculations). - .gif.bak (the Gaussian 09 file used to generate the contour maps, i.e. the Highest Occupied Molecular Oribital [HOMO] maps and the Lowest Unoccupied Molecular Orbital [LUMO] maps). - .mol2 (input file for Schrödinger used to generate the docking results). The Gaussian 09 files can be opened by readers using a non-proprietary software, such as ORCA, mentioned previously in the methods. The input files were obtained using Gaussview 6.1, the visualization program of Gaussian 09. Data are available under the terms of the Creative Commons Zero “No rights reserved” data waiver (CC0 1.0 Public domain dedication).
