# Supplementary material for: Understanding the high electronic quantum similarity of a series of ligands used as inhibitors of the SARS-CoV-2 virus by molecular mechanics and density functional theory approaches
Source: F1000Res. 2024 Feb 6;12:669. Originally published 2023 Jun 14. [Version 2] doi: 10.12688/f1000research.127061.2 (PMC12873539; doi:10.12688/f1000research.127061.2)
Supplement: Peer review history

# Reviewer report: Reviewer response for version 2

**DOI:** 10.5256/f1000research.161311.r449741

**Published:** 2026-02-04

**Authors:** Sharvan Sehrawat

**Affiliations:** 1 Biological science, Indian Institute of Science Education and Research Mohali, Mohali, Punjab, India

While the preliminary in-silico data is valuable, it has to be supported by biological analysis. One can easily find interactions using in-silico analysis but the physiological relevance of such data is questionable. Without such analyses, the enthusiasm remains always low in interpreting such data. The authors should atleast highlight this point in the conclusion/discussion so that data is not over interpreted.

Is the work clearly and accurately presented and does it cite the current literature?

Yes

If applicable, is the statistical analysis and its interpretation appropriate?

Yes

Are all the source data underlying the results available to ensure full reproducibility?

Yes

Is the study design appropriate and is the work technically sound?

Partly

Are the conclusions drawn adequately supported by the results?

Partly

Are sufficient details of methods and analysis provided to allow replication by others?

Yes

Reviewer Expertise:

VIral Immunology and Immunopathology

I confirm that I have read this submission and believe that I have an appropriate level of expertise to confirm that it is of an acceptable scientific standard, however I have significant reservations, as outlined above.

# Reviewer report: Reviewer response for version 2

**DOI:** 10.5256/f1000research.161311.r207390

**Published:** 2025-02-04

**Authors:** Mohnad Abdalla

**Affiliations:** 1 Shandong University, Jinan, Shandong, China

**Scope and Depth of Analysis**

 The study focuses solely on in-silico approaches, including molecular docking, molecular dynamics (MD), and QSAR studies. While these methods are valuable for preliminary investigations, the analysis lacks sufficient depth and comprehensiveness to support strong conclusions.

**Molecular Dynamics (MD) Limitations**

 The MD simulation was conducted for only 100 ns, which is insufficient to conclusively determine the stability of the protein-ligand complex. Prolonged simulations, typically in the range of 200–500 ns, are necessary to provide a more reliable insight into binding stability and interaction patterns.

**Validation Concerns**

 A critical issue with the manuscript is the absence of experimental validation. In-silico findings, while valuable, must be corroborated with laboratory studies to ensure biological relevance and reliability. Without experimental data, the conclusions remain speculative and lack practical applicability.

**Insufficient Analysis of Protein-Ligand Stability**

 The parameters assessed in the MD and QSAR studies are not sufficient to convincingly demonstrate the stability and efficacy of the protein-ligand complex. Additional analyses, such as free energy calculations and more extensive binding interaction studies, would strengthen the findings.

**Significance and Practical Relevance**

 The study does not provide a clear pathway to translate the in-silico findings into a meaningful biological or therapeutic context. This diminishes its overall impact and relevance.

**Recommendation**

 Given the above concerns, especially the lack of experimental validation and insufficient MD analysis, I recommend rejecting the manuscript in its current form. The authors are encouraged to conduct complementary laboratory experiments and provide more robust in-silico data before resubmitting to a journal.

Is the work clearly and accurately presented and does it cite the current literature?

No

If applicable, is the statistical analysis and its interpretation appropriate?

No

Are all the source data underlying the results available to ensure full reproducibility?

No

Is the study design appropriate and is the work technically sound?

No

Are the conclusions drawn adequately supported by the results?

No

Are sufficient details of methods and analysis provided to allow replication by others?

No

Reviewer Expertise:

structural biology, computational modeling, and biopharmaceutical research.

I confirm that I have read this submission and believe that I have an appropriate level of expertise to state that I do not consider it to be of an acceptable scientific standard, for reasons outlined above.

# Reviewer report: Reviewer response for version 2

**DOI:** 10.5256/f1000research.161311.r300998

**Published:** 2024-07-16

**Authors:** Vikram Dalal

**Affiliations:** 1 Washington University School of Medicine, Saint Louis, USA

The manuscript entitled “Understanding the high electronic quantum similarity of a series of ligands used as inhibitors of the SARS-CoV-2 virus by molecular mechanics and density functional theory approaches” provides crucial insight into chemical reactivity and similarities of a set of reported molecules for RdRP of SARS-CoV-2 using in-silico techniques. The authors' work, which includes molecular docking and quantum mechanics to explore the role of stabilization interactions among protein-ligand complexes, is important to our field. While the manuscript is well-written and the work is performed systematically, a few concerns must be addressed. I recommend a major revision and the following suggestions are essential to enhance the quality of the manuscript:

 1. In the Molecular docking section of Methods, it's crucial to provide details like charges on protein and ligands, grid box size, grid box dimensions, amino acid residues considered for grid generation and number of poses generated during molecular docking. This information is essential for the reader to understand the methodology and results. Authors can refer to the following articles for further details: - (Dhankhar et al.,2020)(Ref-1); (kumari et al.,2022)(Ref-2)

 2. Figures 1 to 9 of the molecular docking illustrations require revisions to highlight the representation and labeling of amino acid residues clearly. Additionally, the figure captions need to be modified to include descriptions of the types and colors designated for the protein, ligand, and interactions. For guidance on these modifications, authors can refer to the cited articles detailing the enhancements of molecular docking figures and captions: - ( (Dhankhar et al.,2020)(Ref-1);kumari et al.,2022)(Ref-2) )

 3. It would be beneficial for authors to provide figures representing the energy gaps of HOMO and LUMO to facilitate the analysis of molecular reactivity. Understanding and comparing HOMO and LUMO plots for ligands can be challenging. For additional details on HOMO and LUMO figures, authors can refer to the following article.": - (Dalal et al.,2021)(Ref-3)

 4. The authors thoroughly investigated a crucial topic regarding the potential inhibitor for SARS-CoV-2 using in-silico techniques. It is important to note that they have yet to delve into the potential candidates identified for SARS-CoV-2 using structure-based drug design. The subsequent articles provided comprehensive reports on the potent molecules by targeting various essential elements of SARS-CoV-2.:- (Kumar et al.,2021)(Ref-4);(Dhankhar et al.,2021)(Ref-5); (Dhankhar et al.,2020)(Ref-1)(kumari et al.,2022)(Ref-2)

 5. Ensure that the manuscript undergoes thorough screening to correct any typos and grammatical errors.

Is the work clearly and accurately presented and does it cite the current literature?

Partly

If applicable, is the statistical analysis and its interpretation appropriate?

Yes

Are all the source data underlying the results available to ensure full reproducibility?

Yes

Is the study design appropriate and is the work technically sound?

Yes

Are the conclusions drawn adequately supported by the results?

Partly

Are sufficient details of methods and analysis provided to allow replication by others?

Yes

Reviewer Expertise:

Structure Biology, Structure based drug designing, Protein X-Ray crystallography, Cryo Electron Microscopy, Infectious disease, Ion channels,

I confirm that I have read this submission and believe that I have an appropriate level of expertise to confirm that it is of an acceptable scientific standard, however I have significant reservations, as outlined above.

## References

[1] : Computational guided identification of novel potent inhibitors of N-terminal domain of nucleocapsid protein of severe acute respiratory syndrome coronavirus 2. *J Biomol Struct Dyn* .2022;40(9) : 10.1080/07391102.2020.1852968 4084-4099 10.1080/07391102.2020.1852968 33251943 PMC7754992

[2] : Promising antivirals for PLpro of SARS-CoV-2 using virtual screening, molecular docking, dynamics, and MMPBSA. *J Biomol Struct Dyn* .2023;41(10) : 10.1080/07391102.2022.2071340 4650-4666 10.1080/07391102.2022.2071340 35510600

[3] : Structure-Based Identification of Potential Drugs Against FmtA of Staphylococcus aureus: Virtual Screening, Molecular Dynamics, MM-GBSA, and QM/MM. *Protein J* .2021;40(2) : 10.1007/s10930-020-09953-6 148-165 10.1007/s10930-020-09953-6 33421024

[4] : Multifunctional inhibitors of SARS-CoV-2 by MM/PBSA, essential dynamics, and molecular dynamic investigations. *J Mol Graph Model* .2021;107: 10.1016/j.jmgm.2021.107969 107969 10.1016/j.jmgm.2021.107969 34237666 PMC8220440

[5] : Screening of Severe Acute Respiratory Syndrome Coronavirus 2 RNA-Dependent RNA Polymerase Inhibitors Using Computational Approach. *J Comput Biol* .2021;28(12) : 10.1089/cmb.2020.0639 1228-1247 10.1089/cmb.2020.0639 34847746 PMC8819516

# Reviewer report: Reviewer response for version 1

**DOI:** 10.5256/f1000research.139529.r191736

**Published:** 2023-12-13

**Authors:** Kiran Bharat Lokhande

**Affiliations:** 1 Dr. D. Y. Patil Biotechnology and Bioinformatics Institute, Pune, Maharashtra, India

Comments: 

**To the Authors:**

**Detailed Methodology:** It would greatly enhance the reproducibility of your study if you could provide more detailed information regarding the protein preparation process. Specifically, please include information about the pH levels used in the methodology.

**PDB ID Citation:** I kindly request that you cite the PDB ID used in this study following the guidelines provided by the RCSB. This will ensure proper attribution of the structural data used in your research.

**Experimental Depth:** The study primarily focuses on molecular docking, which has its limitations. Since a similar study with MD simulation studies was published two years ago, it is advisable to incorporate high-end atomistic simulations to support your docking results and provide additional depth to your research.

**MD Simulations:** To strengthen your findings, consider conducting at least 1 microsecond MD simulations in triplicate. This will help validate the outcomes of your molecular docking experiments.

**Article Suitability:** Given the preliminary nature of the study and the existence of a similar study with MD simulations, I recommend an extensive revision of the article to either provide novel insights or enhance the depth of the research before considering it for publication.

**To the Editor:**

1. I would like to highlight several concerns regarding the manuscript in question:
2. The methodology lacks sufficient detail, particularly regarding the protein preparation process, including the pH levels used.
3. The PDB ID used in the study needs to be cited following RCSB guidelines to ensure proper attribution.
4. The study primarily relies on molecular docking, which may not provide the necessary depth of analysis, especially considering a similar study with MD simulations published two years ago.
5. To address this limitation, the authors should conduct high-end atomistic simulations, such as 1 microsecond MD simulations in triplicate, to strengthen the validity of their findings.
6. In its current form, the article appears to be premature and would benefit from extensive revisions to enhance its suitability for publication.
7. I recommend considering these points in the review process to help the authors improve the quality and depth of their research.

Is the work clearly and accurately presented and does it cite the current literature?

No

If applicable, is the statistical analysis and its interpretation appropriate?

Not applicable

Are all the source data underlying the results available to ensure full reproducibility?

No

Is the study design appropriate and is the work technically sound?

No

Are the conclusions drawn adequately supported by the results?

No

Are sufficient details of methods and analysis provided to allow replication by others?

No

Reviewer Expertise:

Expertise in Structural Bioinformatics and Computer-Aided Drug Discovery

I confirm that I have read this submission and believe that I have an appropriate level of expertise to state that I do not consider it to be of an acceptable scientific standard, for reasons outlined above.

# Response

**Published:** 2024-01-11

**Authors:** Alejandro Morales-Bayuelo

**Affiliations:** Universidad del Sinú, Colombia

Detailed Methodology: It would greatly enhance the reproducibility of your study if you could provide more detailed information regarding the protein preparation process. Specifically, please include information about the pH levels used in the methodology.

 Answer: pH 6.2 to 6.8

 PDB ID Citation: I kindly request that you cite the PDB ID used in this study following the guidelines provided by the RCSB. This will ensure proper attribution of the structural data used in your research.

 Answer: PDB ID: 6M71Download ; MMDB ID: 188116 ; PDB Deposition Date: 2020/3/16.

 Experimental Depth: The study primarily focuses on molecular docking, which has its limitations. Since a similar study with MD simulation studies was published two years ago, it is advisable to incorporate high-end atomistic simulations to support your docking results and provide additional depth to your research. MD Simulations: To strengthen your findings, consider conducting at least 1 microsecond MD simulations in triplicate. This will help validate the outcomes of your molecular docking experiments.

 Answer: All the docking results were supported by molecular dynamic of 20 ns. To verify the molecular stabilization of the ligands on the active site.

 Article Suitability: Given the preliminary nature of the study and the existence of a similar study with MD simulations, I recommend an extensive revision of the article to either provide novel insights or enhance the depth of the research before considering it for publication.

 Answer: The article was revised.

# Response

**Published:** 2024-01-11

**Authors:** Alejandro Morales-Bayuelo

**Affiliations:** Universidad del Sinú, Colombia

The influence of pH (acidity or alkalinity) on the SARS-CoV-2 virus, which causes COVID-19, is an interesting topic. However, it's important to note that SARS-CoV-2 primarily infects and spreads among human cells, and the virus itself doesn't exist independently in the environment for long periods.

 The pH level becomes more relevant when considering how the virus interacts with the human body. The human body maintains various pH levels in different parts, and this plays a role in the virus's ability to infect cells. For example:

 Respiratory Tract: The virus primarily enters the body through the respiratory tract. The pH levels in the respiratory tract can vary, and some studies suggest that certain viruses, including coronaviruses, might be influenced by these pH levels. However, the exact relationship is complex and may not be the sole determinant of infection.

 Cellular Environment: Once the virus enters human cells, the pH of the cellular environment becomes crucial. Inside cells, the virus needs to release its genetic material and replicate. The pH within cellular compartments can affect these processes.

 Immune Response: The body's immune response also involves pH-dependent processes. Immune cells often function optimally within specific pH ranges, and any disruption might influence the ability of the immune system to combat the virus.

 It's important to emphasize that pH is just one of many factors influencing viral infections, and the interplay of these factors is intricate. Factors like temperature, humidity, and the overall health of an individual also contribute to the susceptibility and severity of viral infections.

# Reviewer report: Reviewer response for version 1

**DOI:** 10.5256/f1000research.139529.r178627

**Published:** 2023-12-12

**Authors:** Ramon Carbó-Dorca

**Affiliations:** 1 Institute of Computational Chemistry and Catalysis, University of Girona, Girona, Spain

This study presents in an original way a well-structured study of inhibitors of the COVID virus.

 The authors give a balanced point of view of the problem using several techniques, among others, quantum similarity analysis.

 I have been impressed with the amount of work implemented in the paper.

 I will suggest the publication without changes, except for an editorial revision of the text, just in case there are some incorrections that this referee has not been able to detect.

Is the work clearly and accurately presented and does it cite the current literature?

Yes

If applicable, is the statistical analysis and its interpretation appropriate?

Yes

Are all the source data underlying the results available to ensure full reproducibility?

Yes

Is the study design appropriate and is the work technically sound?

Yes

Are the conclusions drawn adequately supported by the results?

Yes

Are sufficient details of methods and analysis provided to allow replication by others?

Yes

Reviewer Expertise:

Essentially: Quantum Similarity

I confirm that I have read this submission and believe that I have an appropriate level of expertise to confirm that it is of an acceptable scientific standard.

# Reviewer report: Reviewer response for version 1

**DOI:** 10.5256/f1000research.139529.r207285

**Published:** 2023-11-22

**Authors:** Ahmed Metwaly

**Affiliations:** 1 Al-Azhar University, Cairo, Egypt

I have thoroughly reviewed the manuscript entitled "Understanding the high electronic quantum similarity of a series of ligands used as inhibitors of the SARS-CoV-2 virus by molecular mechanics and density functional theory approaches" submitted to F1000Research. 

 The authors have undertaken a commendable effort to employ molecular mechanics and density functional theory approaches to analyze the ligands' potential as inhibitors of the SARS-CoV-2 virus.

 However, my primary concern lies in the absence of experimental validation, particularly *in vitro* studies, to support the computational findings. Despite the availability of the examined compounds and RdRp binding kits, the manuscript lacks crucial experimental data that would enhance the credibility and transnational impact of the study. Experimental validation is essential to confirm the practical applicability of the proposed ligands as SARS-CoV-2 inhibitors.

 Given the current limitations, I recommend rejecting the manuscript in its present form. I believe that the inclusion of *in vitro* experimental data would significantly strengthen the study and its potential impact on drug development for COVID-19. I encourage the authors to consider conducting and incorporating such experiments in the next submission.

Is the work clearly and accurately presented and does it cite the current literature?

Partly

If applicable, is the statistical analysis and its interpretation appropriate?

Not applicable

Are all the source data underlying the results available to ensure full reproducibility?

No

Is the study design appropriate and is the work technically sound?

No

Are the conclusions drawn adequately supported by the results?

Partly

Are sufficient details of methods and analysis provided to allow replication by others?

Partly

Reviewer Expertise:

Drug Discovery

I confirm that I have read this submission and believe that I have an appropriate level of expertise to state that I do not consider it to be of an acceptable scientific standard, for reasons outlined above.
